# Artificial intelligence in spine care: A scoping review of diagnostic applications

**DOI:** 10.1371/journal.pone.0352200

**Published:** 2026-07-28

**Authors:** Victoria A. Bensel, Anne Habeck, Marcda Hilaire Brunot, Eleni-James Becton, Monika Ray, Alexandria L. Brackett, Anthony J. Lisi

**Affiliations:** 1 Department of Biomedical Informatics and Data Science, Yale School of Medicine, New Haven, Connecticut, United States of America; 2 VA Connecticut Healthcare System, West Haven, Connecticut, United States of America; 3 Bristol, Connecticut, United States of America; 4 Private Practice, Jacksonville, Florida, United States of America; 5 Department of Clinical Research, The University of Jamestown, Jamestown, North Dakota, United States of America; 6 Department of Internal Medicine, School of Medicine, University of California Davis, Sacramento, California, United States of America; 7 Center for Healthcare Policy and Research, University of California Davis, Sacramento, California, United States of America; 8 Harvey Cushing/John Hay Whitney Medical Library at Yale University, New Haven, Connecticut, United States of America; National Institute of Child Health and Human Development (NICHD), NIH, UNITED STATES OF AMERICA

## Abstract

**Background:**

Artificial intelligence (AI) is increasingly used to enhance diagnostic accuracy, automate image interpretation, and support clinical decision-making. In the field of spine care, applications include MRI and CT-based detection of lumbar disc degeneration, spinal stenosis, vertebral fractures, and axial spondyloarthritis, as well as emerging symptom-based and multimodal diagnostic tools. However, evidence remains dispersed across modalities and conditions, and the quality and clinical readiness of AI systems vary. This scoping review maps current AI applications for diagnosing spinal disorders and identifies gaps for future research and clinical translation.

**Methods:**

This review followed Joanna Briggs Institute (JBI) and PRISMA-ScR guidelines. Ovid MEDLINE, AMED, Embase, Cochrane CENTRAL, Web of Science, and Scopus were searched from January 2019 to December 2024. Eligible studies were mapped according to AI methodology, diagnostic target, data source, and validation approach, and were required to involve human participants, include sufficient methodological detail, and published in English peer-reviewed journals. No geographic restrictions were applied. Data was extracted on study design, AI methodology, diagnostic target, validation approach, and usability. Methodological quality was assessed using a 19-point scoring system covering study design, reporting clarity, data validation, and feature selection.

**Results:**

Forty-six studies met the inclusion criteria, conducted primarily in Asia and Europe, with two studies from North America and one from South America. Most investigations were retrospective, imaging-based deep learning models applied to MRI or CT for detecting disc herniation, lumbar spinal stenosis, modic changes, vertebral fractures, and sacroiliitis. Several studies used prospective designs or external validation. Diagnostic performance was generally high across imaging models, with many studies describing accuracy that approached or matched clinician benchmarks, particularly in sacroiliitis classification, disc disease detection, and stenosis grading. Methodological scores ranged from 7.5 to 17.5 out of 19, with recurrent weaknesses in handling missing data, feature selection, and data element validation.

**Conclusion:**

This review maps a growing body of literature on AI applications for diagnosing spinal disorders, with studies most frequently reporting favorable performance for MRI- and CT-based detection of degenerative and inflammatory conditions. Evidence remains preliminary and heterogeneous.

## Introduction

Spinal disorders are among the leading causes of disability worldwide, contributing substantially to pain, reduced quality of life, and healthcare expenditures [1–3]. Conditions such as low back pain, spinal stenosis, spondylolisthesis, ankylosing spondylitis, and vertebral fractures represent a diverse spectrum of pathologies that often present with overlapping symptoms [4]. Accurate diagnosis is critical, as treatment pathways vary widely depending on the underlying etiology, disease severity, and patient comorbidities [5]. However, diagnostic evaluation in spine care remains complex and often inconsistent [6].

Current diagnostic approaches rely heavily on clinical examination and imaging modalities such as plain radiographs, magnetic resonance imaging (MRI), and computed tomography (CT) [7,8]. While these tools are indispensable, they are also limited by subjective interpretation, inter-observer variability, and frequent discrepancies between radiographic findings and clinical symptoms [9–11]. For example, asymptomatic degenerative changes are common on MRI, which complicates the differentiation between incidental findings and clinically meaningful pathology [12,13]. This variation in diagnostic accuracy among practitioners can ultimately lead to misdiagnosis, delayed treatment, and unnecessary interventions.

Additionally, the growing demand for spine-related imaging has further highlighted inefficiencies in traditional diagnostic pathways. Radiological services face increasing workload pressures, and primary care clinicians may lack the specialized expertise required to interpret complex spinal findings [14–16]. Consequently, there is a pressing need for early and precise diagnosis to guide appropriate management and avoid overtreatment, particularly given the global rise in musculoskeletal disability [17,18].

Artificial intelligence (AI) offers potential solutions to these challenges by enhancing diagnostic precision, standardizing interpretation, and integrating multimodal data sources [19–21]. Machine learning algorithms and deep learning models can be applied to spinal imaging to automatically classify pathologies, quantify structural changes, and detect subtle abnormalities [22,23]. Natural language processing tools are increasingly being used to extract diagnostic insights from electronic health records and radiology reports [24,25]. Predictive models trained on large datasets also show promise for risk stratification and early disease detection [26,27]. Despite this potential, the current evidence base is fragmented, with studies varying widely in methodology, diagnostic targets, and reporting standards [28–30].

Given these challenges and opportunities, this scoping review aims to provide an overview of AI-based diagnostic applications in spine care, summarize key findings, and highlight areas where further validation and clinical integration are required.

## Methods

This scoping review was conducted in accordance with the Joanna Briggs Institute (JBI) methodology for scoping reviews and follows the Preferred Reporting Items for Systematic Reviews and Meta-Analyses extension for Scoping Reviews (PRISMA-ScR) guidelines.

### Search strategy

A comprehensive search strategy was developed in collaboration with a medical librarian (AB) to identify relevant studies on the application of AI in spine diagnostics. The search covered literature published between January 1, 2019, and December 31, 2024. This time window was selected to capture the period of rapid expansion in clinical AI research following the widespread adoption of deep learning methods in medical imaging, which accelerated substantially from 2019 onward [31]. Electronic databases searched included Ovid MEDLINE, AMED, Embase, Cochrane CENTRAL, Web of Science, and Scopus. Search terms combined AI-related concepts (e.g., “machine learning,” “deep learning,” “neural networks,” “natural language processing”) with spine care and diagnostic terms (e.g., “diagnosis,” “classification,” “detection,” “prediction,” “imaging”). Controlled vocabulary was also used when applicable. The only filter applied was to limit the publication years. All identified citations were deduplicated using the Yale University Harvey Cushing/John Hay Whitney Medical Library Reference Deduplicator tool prior to importation and then screening in Covidence. See S1 File for the full search string.

### Source evidence selection

After de-duplication, three independent reviewers (VB, MB, AH) screened titles and abstracts for eligibility. Full-text articles were retrieved for studies that met the inclusion criteria or when eligibility was uncertain. Discrepancies were resolved by discussion among primary reviewers (VB, MB, AH). When consensus could not be reached, a third reviewer served as a tiebreaker to make the final inclusion decision. Third reviewer adjudication was needed occasionally throughout the screening process. Cohen’s kappa was not calculated as reviewers were not assigned in fixed pairs; however, an overall agreement rate of >85% was achieved across screening and extraction stages. The study selection process was documented in a PRISMA flow diagram.

### Inclusion/exclusion

Studies were eligible if they examined AI applications for diagnostic purposes in spine care (Table 1). Eligible studies involved human participants and compared AI-based diagnostic approaches either to non-AI methods (e.g., clinician interpretation) or to accepted diagnostic benchmarks (e.g., radiology standards, pathology-confirmed findings). Both imaging-based and non-imaging diagnostic applications were included. The unifying criterion across all included studies was the application of an AI model to a diagnostic task in spine care, regardless of input data modality. Exclusion criteria were studies focused exclusively on administrative, financial, or non-clinical applications of AI; animal or cadaveric studies; and reports lacking a clinical decision-making component.

**Table 1:** Eligibility criteria and their rationale.

Eligibility Criteria and Variable	Rationale
Peer Reviewed	Ensures scientific rigor and credibility
Published between January 1^st^, 2019-December 31^st^, 2024.	Reflects recent developments in the field
English Language	Accessible to the research team
Involved human participants	Focuses on clinical relevance
Compared AI-based approaches to non-AI methods or benchmarked against standards	Allows for meaningful evaluation of AI performance
**Exclusion Criteria**	
Studies focused on administrative, financial or non-clinical applications of AI	Not relevant to patient care
Studies on animals or cadavers	Not applicable to live clinical settings

### Data extraction

A sample data extraction was performed independently by two reviewers (AH, VB) using a structured extraction template, achieving an > 85% level of agreement, with remaining studies divided between the two extractors. Extracted data included study characteristics (author, year, country, design, and population), AI model type, input data source (e.g., MRI, CT, X-ray, clinical notes), comparator (e.g., radiologist or gold standard), diagnostic task (e.g., detection, classification, grading), performance metrics (e.g., sensitivity, specificity, area under the curve (AUC)), validation approach, and key findings related to diagnostic accuracy, clinical utility, and implementation considerations.

### Data analysis

Extracted data were synthesized narratively and summarized in tabular form. Studies were categorized based on diagnostic tasks, AI technology type, and clinical application area. Comparisons between AI and conventional diagnostic methods were emphasized, focusing on diagnostic performance, interpretability, workflow integration, and research gaps were identified. The included studies demonstrated substantial heterogeneity in model architecture, input data sources, outcome definitions, and validation approaches. Quantitative synthesis was therefore not appropriate.

### Quality assessment

While formal quality appraisal is not required in scoping reviews, we conducted a structured quality assessment to contextualize the methodological rigor of included studies. We developed a custom checklist adapted from the APPRAISE-AI tool [32], and the TRIPOD-AI extension [33]. This tool evaluates key elements of AI-based clinical research, including data representativeness, transparency, bias mitigation, model performance, and reporting practices (Table 2).

**Table 2:** Structured quality appraisal domains and criteria adapted from APPRAISE-AI and TRIPOD-AI.

Domain	Criterion	Definition
Data	Sample Size and Representativeness	Does the manuscript provide a statistically sufficient dataset and report key demographic characteristics? If demographic data are unavailable, does it acknowledge limitations and discuss generalizability of findings?
	Handling of Missing Data and Class Imbalances	Does the manuscript describe how missing data were handled (e.g., imputation, removal) and whether class imbalances were addressed?
	Data Source Transparency	Does the manuscript clearly describe data sources, their origin, collection methods, and preprocessing steps, including any access limitations?
	Bias Identification and Documentation	Does the manuscript identify potential dataset biases and describe mitigation techniques (e.g., re-sampling, re-weighting)? If biases remain, are implications discussed?
	Fair Data Practices	Does the manuscript report implementation of fair data handling practices (e.g., federated learning, confidential computing, or special protections for vulnerable populations)?
	Data Availability	Does the manuscript confirm accessibility of the dataset and specify storage methods?
	Privacy and Consent Compliance	Does the manuscript demonstrate compliance with privacy regulations and informed consent protocols?
	Financial and Ethical Disclosures	Does the manuscript disclose funding sources and potential conflicts of interest?
	Data Elements Validation	Does the manuscript verify data validity at the source and confirm accuracy for standardized codes (e.g., ICD10, PCS codes)?
	Data Dictionary	Does the manuscript provide a clear definition of all variables and coding lists (e.g., ICD10/PCS) where applicable?
Model	Model Selection Justification	Does the manuscript justify the choice of model type (e.g., decision tree, neural network) based on theoretical or empirical evidence?
	Target Variable Definition and Measurement	Does the manuscript clearly define the predicted outcome, including measurement methods and any transformations applied?
	Baseline Model Comparisons	Does the manuscript compare performance against prior models, statistical baselines, or human experts, and justify if no baseline was used?
	Feature Selection Methods	Does the manuscript describe the feature selection process, including the role of domain knowledge or automated techniques (e.g., principal component analysis (PCA), least absolute shrinkage and selection operator (LASSO))?
	Model Specifications	Does the manuscript report model structure, including hyperparameters and tuning methods? For neural networks, are layers, activation functions, and optimization techniques specified?
	Bias Identification and Mitigation	Does the manuscript evaluate bias in model predictions and provide evidence (e.g., calibration bands, Hosmer–Lemeshow plots) of performance across subpopulations?
	Comprehensive Performance Metrics	Does the manuscript report predicted vs. observed outcomes, measures to mitigate overfitting, subpopulation-level performance, and uncertainty estimation (i.e., generalizability)?
	Data Splits	Does the manuscript clearly define training, validation, and test set allocations, and provide justification for chosen split ratios? If cross-validation was not performed, is a valid explanation provided?
	Analytical Packages Mentioned	Does the manuscript list all software and libraries used for model development?

Each item was scored on a 0–1 scale (0 = not met, 0.5 = partially met, 1 = fully met), with results summarized to guide interpretation of study quality. A score of 0.5 was assigned when a criterion was addressed but incompletely, such as when a study acknowledged missing data without describing how it was handled. Borderline cases were discussed between reviewers, and the 0.5 designation was applied consistently across studies. To ensure consistency in scoring, two independent reviewers assessed all studies, and a third reviewer was available when discrepancies occurred.

## Results

**Fig 1:**
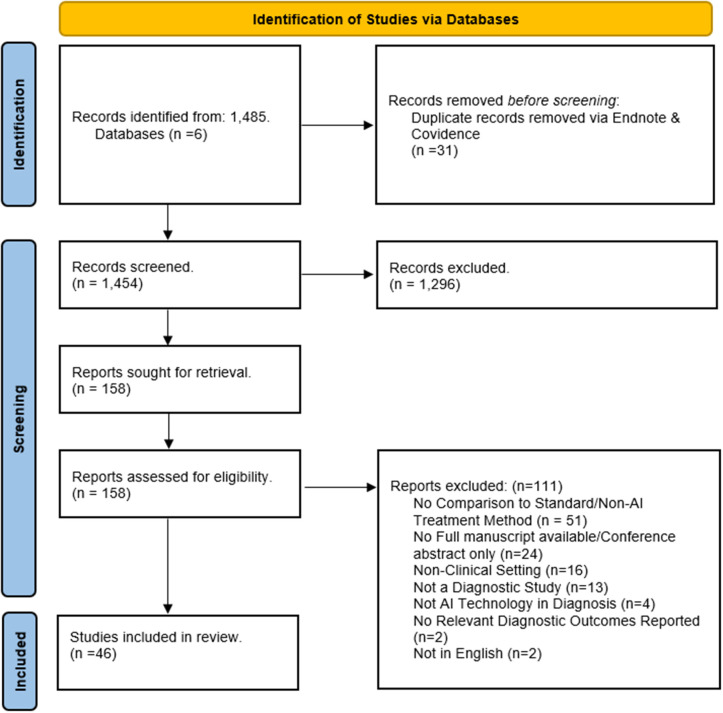
PRISMA flow diagram of study selection for AI diagnosis applications in spine care.

A total of 1,485 manuscripts were identified through searches across six databases. After removing 31 duplicates, 1,454 studies were screened by title and abstract. Ultimately, 46 studies met all criteria and were included in the final review (Fig 1).

Forty-six studies met inclusion criteria (Table 3), published between 2019 and 2025 and conducted predominantly in Asia (n = 24), followed by Europe (n = 14), North America (n = 3), South America (n = 1), and the Middle East (n = 2), with two multinational cohorts. The majority of studies were retrospective and imaging-based diagnostic evaluations using deep learning architectures applied to lumbar MRI or CT for conditions such as disc herniation, spinal stenosis, modic changes, vertebral fractures, and axial spondyloarthritis [34–38]. Several prospective studies were also identified [39–42], along with cross-sectional or diagnostic accuracy designs [43,44]. Across studies, artificial intelligence was used primarily for detection, segmentation, classification, and grading of spinal pathology on imaging.

**Table 3:** Overview of articles included in the scoping review (N = 46).

Author, Year	Country	Study Design	Population Studied	Type of AI Used	Purpose of the AI (e.g., detection, classification, grading of pathology, triage)	Type of diagnosis (e.g., imaging-based, symptom-based, composite)	Methodological Rating (x/ 19)
Abdollah et al. 2021 [60]	Canada	Case–control study	Adults with chronic low back pain (LBP) and age/sex/weight-matched healthy controls	Random Forests Algorithm used for feature selection and classification. A custom MATLAB-based texture analysis software was used to construct gray-level co-occurrence matrices (GLCM).	Classification/differentiation of image texture features in persons with and without LBP	Imaging-based	12
Athertya et al. 2019 [69]	India	Cohort study	Adults undergoing lumbar MRI for evaluation of low back pain and modic changes	Random Forest, SVM, k-Nearest Neighbor, Naive Bayes	Detection and classification of modic changes (type I, type II, normal) in lumbar vertebrae	Imaging-based	16.5
Badahman et al. 2024 [44]	Saudi Arabia	Diagnostic Accuracy Study (multi-stage: case series, Delphi, cross-sectional)	Adult patients with low back pain (LBP), specifically those with or without lumbar disc herniation	Therapha (v1.3.5), a web-based Clinical Decision Support System (CDSS) incorporating a supervised AI model and a conversational chatbot interface.	Detection/classification of lumbar disc herniation as clinical decision support for triage/diagnosis	Composite: symptom-based (structured clinical input) compared to imaging (MRI)	16.5
Bordner et al. 2023 [59]	France, Spain	Diagnostic Accuracy, Deep Learning Model Evaluation	Adults with chronic inflammatory back pain, assessment of sacroiliitis/axial spondyloarthritis	Deep learning model using an end-to-end Mask Region-based Convolutional Neural Network (Mask-RCNN) with a ResNet-50 backbone, pretrained on ImageNet. Two independent U-Nets were used for automatic bone segmentation (sacrum and iliac) during training.	Detection/classification of bone marrow edema (BME) and active sacroiliitis by Assessment of Spondyloarthritis International Society (ASAS) criteria	Imaging-based	17.5
Bressem et al. 2023 [45]	International (PROOF): 29 countries	Diagnostic Accuracy, Deep Learning Model Evaluation	Adults with axial spondyloarthritis (axSpA) (radiographic and non-radiographic)	Two-stage deep learning pipeline: (1) 3D U-Net for MRI denoising, artifact reduction, and image homogenization; (2) 3D dual-encoder ResNet-101 classification network for inflammatory and structural change detection; Grad-CAM for visual explainability; code publicly available on GitHub	Detection/classification of definite radiographic sacroiliitis (for axSpA diagnosis)	Imaging-based	17.5
Bressem et al. 2021 [74]	Germany	Retrospective	Adults with clinical suspicion of axial spondyloarthritis (axSpA)	Convolutional neural network (ResNet-50) pretrained on ImageNet; transfer learning with mix-up, label smoothing, and progressive re-sizing; Grad-CAM for explainability.	Detection of active inflammatory and structural changes in sacroiliac joints indicative of axSpA	Imaging-based	17.5
Chalhoub et al. 2024 [63]	Lebanon, France, United States (USA)	Prospective	Patients with various spinal pathologies (disc disease, sagittal malalignment, scoliosis, stenosis, spondylolisthesis, vertebral fracture, facet joint syndrome)	Generative AI language model (ChatGPT-3.5, OpenAI)	Diagnosis and management recommendation for spinal pathologies	Composite: symptom- and imaging-based (patient form included both)	8
Chen et al. 2024 [46]	China	Retrospective	Patients with thoracolumbar vertebral compression fractures (VCFs), both acute and chronic.	Two-stage deep learning pipeline: (1) 3D V-Net (fully convolutional encoder-decoder) for automatic vertebral body segmentation; (2) CNN classifiers: ResNet18, ResNet50, ResNet152, DenseNet121, DenseNet169.	Segmentation of fractured vertebrae and classification as “fresh” (acute) or “old” (chronic)	Imaging-based	16.5
Cheung et al. 2023 [35]	China	Retrospective cohort	Adults (>18 yrs), population-based, without prior spine surgery, marked deformity, or spinal tumor, undergoing lumbar MRI for disc degeneration	Two-stage CNN pipeline (EDPP-Flow): (1) MRI-SegFlow — unsupervised deep learning segmentation of vertebral body and IVD; (2) modified VGG-M CNN (encoder + two fully connected classifier layers) for progression prediction.	Prediction of 5-year progression of endplate defects (Schmorl’s node, high intensity zone (HIZ), modic changes) from baseline MRI	Imaging-based	17
Dorfner et al. 2024 [56]	PROOF: 29 countries	Retrospective	Patients with axial spondyloarthritis (axSpA), both radiographic and non-radiographic	Two-stage pipeline: (1) U-Net CNN for automated segmentation/bounding box of sacroiliac joints; (2) ResNet-50 pretrained on ImageNet-1k for binary classification.	Detection/classification of radiographic sacroiliitis; prediction of progression in axSpA	Imaging-based	17
Faleiros et al. 2020 [61]	Brazil	Retrospective	Active inflammatory sacroiliitis related to axial spondyloarthritis (axSpA)	Support Vector Machine (linear kernel), Multilayer Perceptron (MLP; 1 hidden layer, 231 neurons, learning rate 0.3, momentum 0.2, 500 epochs), and Instance-Based Algorithm (IBk; k = 1,3,5).	Detection of active inflammatory sacroiliitis	Imaging-based	15.5
Gao et al. 2021 [47]	China	Retrospective	Intervertebral disc (IVD) degeneration (primary indications for lumbar MRI were disc herniations and spinal stenosis)	Four CNNs (VGG-M, VGG-16, GoogLeNet, ResNet-34) each trained with and without the proposed PPR strategy.	Detection of IVD.	Imaging-based	17
Gao et al. 2022 [34]	USA	Retrospective	Modic changes (MCs)	Two-stage deep learning pipeline: (1) V-Net CNN for vertebral body segmentation from T1-weighted sagittal images; (2) modified 2D V-Net encoder-decoder for binary MC detection/segmentation from stacked T1 + T2 images.	Detection of modic changes.	Imaging-based	16.5
Georgiev et al. 2023 [40]	Bulgaria	Prospective	Lumbar spine stenosis (LSS)	CoLumbo v2.0: a commercial pre-clinical deep learning software (CNN-based) for lumbar spine MRI analysis (Smart Soft Healthcare).	Detection of LSS.	Imaging-based	16
Hartley et al. 2024 [75]	United Kingdom	Prospective	Low back pain (LBP)	Two-stage pipeline: (1) HigherHRNet CNN (bottom-up pose estimation, pretrained on MS-COCO, 250,000 person instances) extracting ankle, hip, and neck key points from standard video; (2) feedforward neural network classifier with batch normalization, ReLU activation, and binary cross-entropy loss trained on 9 movement features (standard and novel spinal angle statistics) and 7 PROMs.	Automated clinical classification of non-specific low back pain	Imaging-based	17.5
Jans et al. 2021 [39]	Belgium	Prospective	Sacroiliitis	Deep learning–based MRI-to-CT image synthesis using a 3D U-Net architecture (BoneMRI Pelvic Region v1.1, MRIguidance).	Detection of erosions, sclerosis, and ankylosis of the SI joints.	Imaging-based	13.5
Ke et al. 2024 [65]	China	Retrospective	Lumbar degenerative diseases (lumbar spondylolisthesis, lumbar disc herniation, lumbar spinal stenosis)	PP-YOLOv2 object detection algorithm (transfer learning, Baidu PaddlePaddle).	Identification of lumbar disc herniation and/or lumbar spondylolisthesis.	Imaging-based	17
Krabbe et al. 2024 [43]	Denmark	Prospective cross-sectional	Structural lesions (erosion, sclerosis, ankylosis) of sacroiliac joints in patients with spondyloarthritis	Deep learning MRI-to-CT synthesis (BoneMRI V1.4, MRIguidance).	Identification of structural lesions of the sacroiliac joints amongst patients with previously diagnosed axial spondyloarthritis.	Imaging-based	15.5
Lagerstrand et al. 2022 [76]	Sweden	Retrospective	Non-specific low back pain; disc fissures	Random forest algorithm (100 trees; scikit-learn v0.23.2).	Identification of painful disc fissures that extend into the outer layers of the annulus fibrosus.	Imaging-based	16.5
Lee et al. 2021 [49]	Korea	Retrospective	Bone marrow edema of sacroiliac joint in patients diagnosed with axial spondyloarthritis (axSpA)	ResNet18 CNN (transfer learning, ImageNet pre-trained).	Identification and classification of bone marrow edema in patients with axSpA.	Imaging-based	15.5
Lee et al. 2023 [48]	Korea	Retrospective	Sacroiliitis associated with axial spondyloarthritis (axSpA)	DenseNet121 CNN (201 layers; unsupervised transfer learning).	Identification of sacroiliitis from X-ray images.	Imaging-based	16
Lehnen et al. 2021 [73]	Germany	Retrospective	Degenerative changes of lumbar spine	U-Net-based CNN (CoLumbo, SmartSoft Ltd.) with ResNet-50-like feature extractor.	Identification of degenerative changes of the lumbar spine on MR images.	Imaging-based	17.5
Liawrungrueang et al. 2024 [51]	Thailand	Retrospective	Grading of lumbar intervertebral disc degeneration	CNN with YOLO (You Only Look Once) architecture. Input: sagittal T2-weighted lumbar spine MRI (320 × 320 pixels, 1.5T and 3T).	Identification of lumbar intervertebral disc degeneration on MR images.	Imaging-based	17.5
Lim et al. 2022 [52]	Singapore	Retrospective	Grading of lumbar spinal stenosis	Two-stage CNN (Spine AI; publicly available via GitHub). Primary CNN detects region of interest; secondary CNN grades stenosis.	Identification and grading of lumbar spinal stenosis.	Imaging-based	16.5
Lin et al. 2022 [41]	China	Prospective	Detection of active inflammatory sacroiliitis in axial SpA	U-net (CNN with attention gates); two models: one trained on original grayscale STIR MRI, one on “fake-colour” RGB images (preceding/current/subsequent slices mapped to R/G/B channels).	Identification of active inflammatory sacroiliitis in short tau inversion recovery sequence MRI.	Imaging-based	12
Lin et al. 2024 [42]	China	Prospective	Grading of sacroiliitis.	Three-model Attention U-net pipeline: (1) pretrained sacroiliitis detection model (fake-colour images; from Lin 2022); (2) BME segmentation model (grayscale, binary labels); (3) SI region segmentation model (multi-label: background/sacrum/ilium). Frangi filter for reference vessel detection.	Grading of sacroiliitis using Spondyloarthritis Research Consortium of Canada (SPARCC) scoring system on MR images.	Imaging-based	8
Liu et al. 2024 [68]	Germany	Retrospective	Identifying calcified lumbar disc herniation (CLDH).	Three CNN classification models compared: ResNet-34 (best performer; 33 conv layers, residual skip connections, pretrained on ImageNet, fine-tuned), DenseNet-121, and MobileViT_s.	Identification of calcified intervertebral disc segments.	Imaging-based	11
Liu et al. 2024 [67]	China	Retrospective	Ankylosing spondylitis (AS)	Two novel CNN models combining spatial and frequency domain features: FRNet (frequency spectrum + radiomics) and PRNet (phase spectrum + radiomics; best performer).	Diagnose sacroiliitis.	Imaging-based	12
Liu et al. 2024 [66]	China	Retrospective	Modic changes (MCs)	Two-network pipeline: SSD (Single Shot Multibox Detector, VGGNet-based) for lesion localization, followed by ResNet18 (17 conv layers, pretrained on ImageNet, fine-tuned) for MC type classification.	Classify modic changes on intervertebral MRI images.	Imaging-based	13
Miyo et al. 2023 [77]	Japan	Observational Study	LSS	Deep-learning reconstruction (DLR; Advanced intelligent Clear-IQ Engine, Canon Medical Systems) applied to unenhanced lumbar CT images for noise reduction and image quality enhancement.	Improve lumbar CT image quality and interobserver agreement in LSS Assessments	Imaging-based	8
Nigru et al. 2024 [72]	Italy	Retrospective	Patients with spinal disorders/low back pain undergoing lumbar MRI	SpineNetV2. Trained on GENODISC (12,018 discs, multi-centre European) and Oxford Whole Spine datasets.	Grading/classification of 11 lumbar disc radiological features	Imaging-based	7.5
Ono et al. 2023 [53]	Japan	Retrospective	Patients with osteoporotic lumbar vertebral fractures (OLVF)	Two-stage pipeline: YOLOv5x for automatic vertebral body detection/cropping from lateral lumbar radiographs, followed by an ensemble of three CNNs (ResNet-50, DenseNet-161, ResNeXt-50; all pretrained on ImageNet) for 3-class classification.	Classification of vertebrae as normal, old OLVF, or fresh OLVF	Imaging-based	12.5
Redeker et al. 2024 [62]	Germany	Retrospective	Patients with chronic back pain, with and without axial spondyloarthritis (axSpA)	Random forest classifier (ensemble of 500 decision trees; variables per node = √total input variables).	Diagnostic classification: axSpA vs non-axSpA	Composite (clinical, laboratory, imaging)	13
Roels et al. 2023 [57]	Belgium	Prospective	Patients with axial spondyloarthritis (SpA), postpartum women, healthy controls	Fully automated multi-stage CNN pipeline: (1) EfficientDet CNN; (2) U-Net CNN (trained on pseudo-labels from ilastik); (3) ResNet18 CNN.	Detection and prediction of bone marrow edema (BME) on SIJ MRI	Imaging-based	13
Seo et al. 2023 [78]	Republic of Korea	Retrospective	Adult patients undergoing cervical spine MRI for clinical indications	Deep learning-based reconstruction (DLR) using a variational network applied to T2-weighted Dixon MRI of the cervical spine.	Acceleration of MRI acquisition while maintaining/improving quality and lesion detectability	Imaging-based	9.5
Shahzadi et al. 2023 [50]	Pakistan, Spain, Mexico, Angola, Republic of Korea	Retrospective	Patients with low back pain/ LSS	Custom CNN for 4-class classification of lumbar foraminal stenosis (normal, mild, moderate, severe).	Detection and grading of lumbar spinal stenosis (LSS) severity	Imaging-based	9.5
Soin et al. 2022 [70]	United States	Prospective	Patients with chronic spinal pain (low back pain, sacroiliitis, post-laminectomy syndrome, radiculopathy)	Decision tree machine learning algorithm.	Predict diagnosis of spinal pain condition from patient-reported data	Symptom-based, diagnostic classification	9.5
Su et al. 2022 [54]	China	Retrospective	Patients with low back pain undergoing lumbar MRI evaluation	Multi-task classification network	Automated grading of lumbar disc herniation (LDH), lumbar central canal stenosis (LCCS), and lumbar nerve root compromise (LNRC)	Imaging-based	11
Tang et al. 2024 [79]	China	Prospective,	Patients with suspected degenerative lumbar spine disease undergoing MRI	Deep learning-based MRI reconstruction using SubtleMR V2 (Subtle Medical, Menlo Park, USA).	MRI acceleration – reduce scan time while preserving diagnostic quality	Imaging-based	9
Triantafyllou et al. 2023 [71]	Greece, Sweden	Retrospective	Patients undergoing MRI of sacroiliac joints for suspected axial spondyloarthritis (axSpA)	Radiomics models: Logistic Regression, SVM, Random Forest, XGBoost	Detection of active sacroiliitis (bone marrow edema)	Imaging-based	12.5
VanderGraaf et al. 2024 [36]	Netherlands	Retrospective	Patients with low back pain/neurogenic leg pain evaluated for lumbar central canal stenosis (LCCS)	Random forest classifier using quantitative features from deep learning segmentation	Automatic classification of LCCS severity (multiclass and binary)	Imaging-based	10
Yoo et al. 2023 [55]	Republic of Korea	Retrospective	Patients undergoing lumbar spine MRI for suspected degenerative spine conditions	DL-based MRI reconstruction (SwiftMR v2.0.1.0, AIRS Medical). U-net variant; 18 convolutional blocks, 4 max-pooling, 4 upsampling, 4 feature concatenation, 3 convolutional layers in cascade with data consistency enforcement.	Detection and classification of lumbar disc herniation (LDH)	Imaging-based	9
Zhang et al. 2024 [37]	China	Retrospective	Patients with suspected axial spondyloarthritis (axSpA); sacroiliac joint MRI	CNN models, ensemble ML fusion, combined clinical-AI model	Diagnosis of axSpA-related sacroiliitis from MRI	Imaging-based	13.5
Zhang et al. 2023 [58]	China & USA	Retrospective,	Patients with suspected ankylosing spondylitis (AS); sacroiliac joint CTs	nnU-Net segmentation + custom 3D CNN for grading	Automatic segmentation + grading diagnosis of sacroiliitis in AS on CT	Imaging-based	12.5
Zhang et al. 2023 [64]	China	Retrospective	Patients with low back pain undergoing lumbar MRI for suspected lumbar disc herniation (LDH)	Two-stage pipeline on axial T2W lumbar MRI. Stage 1: Faster R-CNN (ResNet-50 backbone; region proposal network + Fast R-CNN detector; 2-class softmax for disc region detection; 4-class softmax for bounding box refinement.	Automated detection and classification of LDH severity on axial lumbar MR images	Imaging-based	12
Zhang et al. 2024 [38]	China	Retrospective	Patients with suspected axial spondyloarthritis (axSpA)	TabNet (attentive interpretable tabular learning; sequential multi-step architecture; feature transformer + attentive transformer with sparsemax activation + feature masking per decision step; batch normalization).	Diagnostic prediction (classification of axSpA vs non-axSpA)	Imaging-based + clinical factor integration	13

### AI modalities and diagnostic purposes

Deep learning models constituted the majority of approaches (Table 3), with convolutional neural networks (CNNs) frequently applied to automated detection and classification tasks [34,45–55]. Several studies integrated segmentation networks such as U-Net [41,45,56–58] or in combination with specialized architectures like Mask-RCNN and EfficientDet [57,59]. Traditional machine-learning approaches, including random forest and support vector machines, were also used for feature-based prediction [60–62], and one study evaluated a generative large language model (LLM) (ChatGPT-3.5) for diagnostic recommendations [63].

Overall, the included studies fell into four broad methodological clusters. The largest comprised deep learning models applied to structural imaging for detection, classification, or grading of pathology. A second cluster used traditional machine learning approaches, including random forest and support vector machines, applied to either imaging-derived features or structured clinical inputs. A third, smaller cluster examined multimodal models that integrated imaging findings with clinical or laboratory variables to improve diagnostic classification, particularly for axial spondyloarthritis. A fourth cluster consisted of a single study evaluating a generative large language model for clinical consultation support. These clusters differed substantially in their input data, model architecture, outcome definitions, and validation strategies, which precluded direct cross-study comparison and informed the decision to conduct a scoping rather than quantitative synthesis.

Models addressed varied diagnostic applications, including sacroiliitis detection [37,39,45,48,49,57,58], lumbar disc disease [35,53–55,64–68], spinal stenosis [36,40,50,52], modic changes [34,46,66,69], and vertebral compression fractures [46,53]. Two studies focused on differentiating acute versus chronic fractures [46,53], while others addressed symptom-based diagnostic classification [70] or combined clinical-imaging models for axial spondyloarthritis [37,38,62].

### Application trends

Imaging-based AI models were frequently reported to show high performance in identifying structural pathology across lumbar and sacroiliac joint disorders. For example, CNN-based systems were described as accurately identifying sacroiliac joint inflammation and structural lesions consistent with axial spondyloarthritis [37,43,45,49,57,71] and distinguishing calcified disc herniations and degenerative lumbar changes [46,66,68,72,73]. Studies evaluating lumbar spinal stenosis and disc herniation reported successful classification and grading [36,40,50,52,54,55,64]. Prognostic imaging models predicted progression of modic changes and other degenerative features [35].

Models based on structured clinical input were reported to perform less consistently [44,70]. though studies examining multimodal approaches combining clinical and imaging features described improvements in diagnostic classification in axial spondyloarthritis [37,38,62]. One LLM-based diagnostic assistant demonstrated potential for clinical consultation support but was not benchmarked against gold-standard criteria [63].

### Diagnostic performance

Across imaging-based systems, studies reported generally high accuracy, with AUCs frequently ≥0.90 in internal testing. Examples include sacroiliitis and BME detection/classification [37,38,45,58,60,74], vertebral compression fracture classification [46,53], multi-feature lumbar grading [54,55,64,72], and lumbar stenosis severity [36]. Several studies reported strong accuracy for disc-related pathology and modic changes [34,35,46–49,51,66,67,69,71,73]. Reconstruction/acceleration work showed preserved lesion detectability with shorter scans [78,79] and improved image quality [77]. One clinical decision support system reported AUC 0.84 for triage of disc herniation [45], while a symptom-driven model matched clinician diagnosis 72% of the time (Table 4) [70].

**Table 4:** Summary table of AI-based diagnostic methods for spinal conditions: outcomes, validation, and usability (N = 46).

Author, Year	Diagnostic Accuracy	Inter- and intra-rater reliability (vs. clinician or gold standard)	Error rates or misclassification rates	Validation method (internal, external, cross-validation)	Impact on Clinicians	Integration into clinical workflow	Usability or interpretability of AI output	Barriers or facilitators to implementation
Abdollah et al. 2021 [60]	No formal diagnostic accuracy metrics (sensitivity, specificity, AUC) were reported. The authors explicitly stated they could not provide diagnostic accuracy estimates because no gold standard existed to define which structure was responsible for the pain.	Not directly reported for AI model, but MRI grading by two experienced raters and a radiology resident; not quantified for AI outputs	Not reported	Internal only. The Random Forests Algorithm was applied to the same dataset used for analysis, with no external validation or cross-validation reported.	Not directly assessed.	Research only; not implemented clinically	Interpretable outputs (Gini index identifies key features), but no user feedback reported	Barriers included small sample size limiting statistical power, use of only a mid-sagittal slice (missing other regions and structures), no test-retest reproducibility across occasions or scanners.
Athertya et al. 2019 [69]	Without data augmentation, the best-performing model (Random Forest with Uniform LBP or LPQ) achieved an accuracy of 81–82%. With SMOTE data augmentation, accuracy improved to 91.7–92%. Sensitivity ranged from 0.612 to 0.802 and specificity from 0.450 to 0.824 depending on the LBP variant. AUROC ranged from 0.56 to 0.84, with Random Forest + Uniform LBP achieving the highest AUROC of 0.84 on test data.	Ground truth labels were provided by a single experienced radiologist. No formal inter- or intra-rater reliability statistics were reported.	Not reported as such; accuracy and AUROC provided	10-fold cross-validation; 80/20 train/test split without replacement for final evaluation	Not reported	Not implemented clinically; proof-of-concept for potential application	No direct report; classifiers and LBP features are generally interpretable	Barriers included a small and imbalanced dataset (only 10 Type I MC cases in raw data), absence of MC Type III in the dataset limiting generalizability, and no external validation. Facilitators included low computational cost relative to competing methods, and the availability of standard open-source tools (MATLAB, WEKA).
Badahman et al. 2024 [44]	AUC of 0.84 (p = 0.001, 95% CI: 0.6–1.0), sensitivity of 88%, specificity of 80%, positive predictive value of 99%, negative predictive value of 27%, positive likelihood ratio of 4.4, and negative likelihood ratio of 0.15.	No formal inter-rater reliability statistic (e.g., kappa) was calculated between Therapha and the expert panel or between raters.	The low negative predictive value of 27% was explicitly attributed to a generalization bias from recruiting only patients with lower back and lower limb symptoms.	Internal validation only. No external validation or cross-validation was performed.	No formal clinician satisfaction, usability testing, or workflow impact study was conducted.	Partially integrated: the software was used prospectively within the hospital setting on the same day as MRI appointments. Each assessment session took approximately 10–15 minutes.	No formal usability study or interpretability assessment was reported.	Barriers reported by the authors included the software being available only in English, a low negative predictive value (27%) due to a study population biased toward LBP with radiculopathy. Facilitators reported included the software being cost-effective and time-efficient (10–15 minutes per assessment).
Bordner et al. 2023 [59]	Internal validation (DESIR baseline): MCC 0.90, sensitivity 93%, specificity 89%, accuracy 90%, AUC 0.98 (95% CI: 0.93–1). DESIR 5-year follow-up: MCC 0.64, sensitivity 77%, specificity 88%, accuracy 86%, AUC 0.90 (95% CI: 0.79–1). DESIR 10-year follow-up: MCC 0.61, sensitivity 61%, specificity 85%, accuracy 81%, AUC 0.80 (95% CI: 0.62–1). External validation (ASAS cohort): MCC 0.62, sensitivity 56% (95% CI: 42–70), specificity 100% (95% CI: 100–100), accuracy 81%, AUC 0.76 (95% CI: 0.57–0.95).	Inter-reader agreement between individual readers ranged from 0.58 to 0.91 in the ASAS cohort, consistent with the authors’ statement that interreader agreement is limited even among experienced physicians.	The model failed to identify the correct number of joints in 7.6% of DESIR baseline, 5.1% at 5 years, 4.5% at 10 years, and 9.8% in the ASAS cohort	Both internal and external validation. Internal validation used three independent DESIR test sets (baseline, 5-year, and 10-year follow-up), all separate from the training set. External validation used the independent international ASAS cohort.	Not specifically quantified; designed to assist non-expert radiologists	Not implemented; proof-of-concept	A model explainability analysis was conducted by a junior radiologist reviewing predictions from one of the 10 Mask-RCNN models for the ASAS cohort.	Barriers reported included a high rate of MRI exclusions due to protocol heterogeneity across centers, particularly mismatched acquisition volumes between T1-weighted and STIR sequences (which the model requires to be synchronized), resulting in a smaller usable dataset. Facilitators reported included the model’s end-to-end design requiring no manual segmentation at inference; the use of an ensemble of 10 networks to improve robustness and correct individual errors.
Bressem et al. 2023 [45]	External test set — inflammatory changes: AUC 0.94 (95% CI: 0.84–0.97), sensitivity 88%, specificity 71%, accuracy 75%. ASAS-compatible changes: AUC 0.88 (95% CI: 0.80–0.95), sensitivity 86%, specificity 76%, accuracy 78%. Structural changes: AUC 0.89 (95% CI: 0.81–0.96), sensitivity 85%, specificity 78%, accuracy 79%. Validation set — inflammatory changes: AUC 0.92, sensitivity 96%, specificity 76%; structural changes: AUC 0.90, sensitivity 95%, specificity 75%	Fleiss kappa for validation set: 0.62 (inflammatory), 0.61 (ASAS-compatible), 0.71 (structural). Fleiss kappa for test set: 0.63 (inflammatory), 0.65 (ASAS-compatible), 0.73 (structural). Consensus of at least 4 of 6–7 raters used as reference standard; 11% of training scans required consensus reading session due to no majority decision	Not isolated; confusion matrices provided, false positives and negatives discussed	Internal validation (73/477 patients randomly selected) and external validation (ASAS cohort, n = 116); training and test datasets fully independent in patients, scanners, and raters	Not formally evaluated in clinical practice; model sensitivity for inflammatory changes (88%) was comparable to non-expert radiologists (83%); model positioned as particularly useful for non-specialized hospitals; clinical utility explicitly stated as requiring further prospective study	Not clinically deployed; research setting only; inference time approximately 18.9 seconds per examination (preprocessing + prediction); authors suggested potential use as classification tool in clinical trials	Grad-CAM activation maps (interpretability of decisions shown visually)	Barriers: Limitations included the absence of a true reference standard, low axSpA prevalence in the test se. Facilitators: Strengths included heterogeneous multicenter training data, a U-Net preprocessing pipeline to normalize images across scanners, an independent external test set.
Bressem et al. 2021 [74]	Validation set: AUC 0.97, sensitivity 88%, specificity 95%, accuracy 90%, kappa 0.79. Independent test set (reader-agreed cases): AUC 0.94, sensitivity 92%, specificity 81%, accuracy 88%, kappa 0.72.	Human interreader kappa 0.53 (agreement 76.9%) on full test dataset; model-reader agreement kappa 0.54–0.57, exceeding human interreader agreement.	Validation: 23/229 misclassified (10%) at balanced cut-off. Test set (reader-agreed): 44/352 misclassified (12.5%); 106/458 cases had reader disagreement with truth unknown.	Internal validation (PROOF, n = 229) and independent external testing (GESPIC, n = 458); fully independent in patients and readers.	Not directly quantified.	Not implemented; research/proof-of-concept	Grad-CAM maps confirmed model correctly focused on sacroiliac joints; binary output with three selectable cut-offs balancing sensitivity and specificity.	Barriers reported by the authors included a reference standard limited to only 2–3 human readers, radiographic heterogeneity introducing labeling uncertainty, and unknown model performance in undiagnosed or non-axSpA populations. Facilitators reported included a large heterogeneous multicenter training dataset from 29 countries, transfer learning with mix-up and label smoothing improving generalizability, an independent external test dataset, and Grad-CAM providing interpretable visual output.
Chalhoub et al. 2024 [63]	Overall diagnostic accuracy 70% (68/97). Misdiagnosis rates by pathology: vertebral trauma 100%, facet joint syndrome 100%, spondylolisthesis 40%, stenosis 40%, scoliosis 40%, disc-related disease 22%.	Not reported	30% (29/97). Mismanagement overall: 53% of cases — missed management 5% (5/97), correct but insufficient plan 10% (10/97), provided only one of multiple valid options 7% (7/97), inappropriate management 5% (5/97).	No validation set or external testing. Single prospective cohort; performance assessed by direct comparison to two-surgeon consensus. No cross-validation or hold-out set.	Not quantified; “advice considered poor in 53% of cases”	Not implemented; research context, proof-of-concept	Output was human-readable, but frequently insufficient or incomplete; no interpretability/justification provided by AI	Barriers authors reported on reliance on outdated training data (cut-off September 2021); inability to interactively question patients; no capacity to perform physical examination; risk of mismanagement in specialist/complex cases. Facilitators reported were that real patient cases used (not vignettes); user-friendly natural language interface; correct diagnosis in 70% and suitable management in 95% of cases overall.
Chen et al. 2024 [46]	DSC 0.90 on validation dataset. Best classification model (ResNet18) — internal validation: accuracy 0.93, sensitivity 0.95, specificity 0.92, precision 0.97, F1 0.95, AUC 0.96. External validation: accuracy 0.88, sensitivity 0.87, specificity 0.88, AUC 0.89. Prospective validation: accuracy 0.88, sensitivity 0.86, specificity 0.90, AUC 0.87.	Not specifically reported for AI, but two radiologists for manual segmentation and consensus diagnosis, disagreements resolved by a third expert.	Not reported in detail; confusion matrices and false negative/positive rates can be inferred from tables	Three-stage: (1) internal validation (n = 59, 8:2 split from South Campus data); (2) independent external validation (n = 34, North Campus, different site, same time period); (3) prospective validation (n = 48, South Campus, Jan–Jun 2024, temporally independent). DeLong tests used for AUC comparisons.	Outperformed both experienced and junior clinicians in AUC/accuracy; enables rapid and automated diagnosis	Not yet implemented; evaluated as a stand-alone tool	Not described in detail; output is binary (fresh/old) classification, with automated segmentation visualization	Barriers reported:retrospective design, selection bias, limited by single-center training data. Facilitators reported: fully automated two-stage pipeline (segmentation + classification).
Cheung et al. 2023 [35]	Schmorl’s nodes: weighted accuracy 89.46 ± 3.71%, sensitivity 89.19 ± 2.70%, specificity 89.72 ± 2.42%, wPPV 89.67 ± 1.20%, wNPV 89.25 ± 4.95%. HIZs: weighted accuracy 91.75 ± 2.48%, sensitivity 93.07 ± 3.96%, specificity 90.43 ± 2.51%, wPPV 90.68 ± 1.19%, wNPV 92.88 ± 3.87%. Modic changes: weighted accuracy 87.51 ± 2.23%, sensitivity 87.93 ± 1.72%, specificity 87.10 ± 1.99%, wPPV 87.20 ± 1.55%, wNPV 87.83 ± 1.23%. No AUC reported.	Not formally reported (no kappa or ICC). Two spine specialists annotated all scans; disagreements were resolved by a third senior surgeon. No quantitative inter-rater agreement statistics provided.	Not reported as explicit misclassification rates. Confusion matrices provided (Fig 2): for Schmorl’s nodes, FP ≈ 85 ± 17, FN ≈ 4 ± 2 per fold; for HIZs, FP ≈ 73 ± 14, FN ≈ 7 ± 4; for Modic changes, FP ≈ 104 ± 16, FN ≈ 7 ± 1. Implied overall error rates ~10–12% based on weighted accuracy figures.	Internal validation only; single cohort (1,152 volunteers) split 85:15 into training and testing sets (4,896 and 864 IVD samples respectively). No independent external validation cohort. Cross-validation approach with reported mean ± SD across folds implied but not explicitly described as k-fold.	Not directly measured; high accuracy and robustness, model outputs interpretable probabilities	Not implemented; validated on research dataset, not clinical workflow	Probability outputs for each pathology, histograms show separation of present/absent; not fully explainable	Barriers reported: no external validation on independent populations; single-cohort development limiting generalizability; highly imbalanced class distribution (Schmorl’s nodes 4.3%, Modic changes 6.7% present) risking reduced reliability. Facilitators reported: weighted metrics (wAcc, wPPV, wNPV) providing more reliable performance evaluation on unbalanced data; pipeline achieved high sensitivity for rare present samples; fully automated end-to-end design from MRI input to progression prediction.
Dorfner et al. 2024 [56]	Anatomy-centered model — GESPIC: AUC 0.899, balanced accuracy 0.821, sensitivity 0.941, specificity 0.700; DAMACT: AUC 0.846, balanced accuracy 0.744, sensitivity 0.851, specificity 0.638; OptiRef: AUC 0.957, balanced accuracy 0.906, sensitivity 0.934, specificity 0.878. Standard model — GESPIC: AUC 0.853, accuracy 0.770; DAMACT: AUC 0.817, accuracy 0.724; OptiRef: AUC 0.947, accuracy 0.850. Anatomy-centred superiority statistically significant for GESPIC (p = 0.0047); not significant for DAMACT or OptiRef. Segmentation U-Net: Dice scores 0.932–0.981 across structures and datasets.	Not formally reported for this study.	Not detailed numerically; false positive “high-risk” predictions tracked for progression	Internal validation on PROOF hold-out set (n = 222, 15%); three independent external test datasets (GESPIC n = 436, OptiRef n = 340, DAMACT n = 163) from different cohorts, countries, and clinical settings. 2-year longitudinal follow-up sub-analysis in GESPIC (n = 251). DeLong’s algorithm for AUC comparisons; McNemar’s test for follow-up comparisons; bootstrapping (1,000 repetitions) for 95% CIs.	Not formally evaluated in a clinical setting.	Not clinically deployed.	Binary output (radiographic sacroiliitis present/absent) with continuous probability score and selectable cut-off. Grad-CAM maps confirmed anatomy-centred model focused specifically on SIJs, avoiding distracting structures (pubic symphysis, hip joints).	Barriers reported: follow-up data limited to 2-year interval (progression timing within that window unknown, longer-term progression unassessed). Facilitators reported: highly diverse training dataset (29 countries); three independent test datasets with different patient characteristics, disease prevalence, and acquisition protocols; direct head-to-head comparison of anatomy-centred vs. standard model.
Faleiros et al. 2020 [61]	Best model (MLP with 6 Wrapper-selected features) — 10-fold cross-validation: sensitivity 100%, specificity 92.3%, accuracy 95.6%, AUC 0.965. External test set: sensitivity 100%, specificity 66.7%, accuracy 80%. Other classifiers on training set: IBk k = 3 achieved highest AUC (0.932); SVM AUC 0.867. No kappa reported.	Not formally quantified.	Training (10-fold CV): 2/46 misclassified (4.4% error rate; both false positives among negatives). External test set: 2/10 misclassified.	10-fold cross-validation on training set (n = 46). External: held-out test set (n = 10, ~ 20% of total dataset), randomly split. Single institution; no truly independent external cohort from a different site.	Not formally evaluated.	Not clinically deployed.	Binary classification output (positive/negative for active sacroiliitis). No visual explainability tools (e.g., Grad-CAM) used.	Barriers reported: Small sample size, single institution, manual image selection and segmentation required. Facilitators reported: first study on SIJ active inflammation on MRI; multiple complementary feature types with systematic feature selection.
Gao et al. 2021 [47]	Best model (ResNet-34 + PPR): overall accuracy 0.860 ± 0.012 vs. 0.760 ± 0.013 without PPR (p < 0.001). Grade-specific improvements with PPR — Grade II: + 15.0% (0.879 vs. 0.729); Grade III: + 14.7% (0.823 vs. 0.676). All four architectures showed >8% overall accuracy improvement with PPR (all p < 0.05). No AUC, sensitivity, or specificity reported.	Not formally quantified.	None reported	Internal validation; 10-time 10-fold cross-validation on a single-centre dataset split by patient (no patient overlap across sets).	Not formally evaluated.	Not clinically deployed.	Five-class grade output (Pfirrmann I–V). t-SNE feature space visualizations demonstrate improved intraclass aggregation and interclass separation with PPR. No patient-facing or clinician-facing explainability tools (e.g., Grad-CAM) reported. Regularization coefficient sensitivity analysis presented.	Barriers reported: Retrospective single-centre design, only T2-weighted sequences evaluated, and selection of regularization coefficients requiring cross-validation tuning. Facilitators reported: PPR strategy significantly improved accuracy across all four CNN architectures, with greatest gains in the most diagnostically challenging grades (II and III); robust three-reader majority consensus reference standard; and t-SNE visualization confirming improved feature separation.
Gao et al. 2022 [34]	Overall MC detection on unseen test set: sensitivity 0.71 (±0.072), specificity 0.95 (±0.022), Cohen’s kappa 0.63 (substantial agreement), detection accuracy 85.7%. Per-type: MC1 sensitivity 0.67/specificity 0.87; MC2 sensitivity 0.67/specificity 0.89; MC3 sensitivity 0.44/specificity 0.83. Vertebral body segmentation Dice 0.882 ± 0.018. No AUC reported.	AI-assisted experiment: initial inter-rater kappa between senior neuroradiologist and junior radiologist κ = 0.52; improved to κ = 0.58 (p < 0.05) with AI assistance. Two senior readers: κ = 0.63 (initial), 0.62 (post-AI, NS). Junior vs. senior MSK radiologist: κ = 0.45 (initial), 0.48 (post-AI, NS). Vertebral body segmentation inter-rater Dice between two research associates: 0.927 ± 0.011.	Not reported as explicit misclassification counts.	Internal validation only; single-centre dataset randomly split into training (n = 50), validation (n = 15), and test (n = 10). No independent external validation. AI-assisted experiment used a separately curated dataset (n = 20) with 4-week washout period; McNemar’s test used for significance.	AI assistance significantly improved junior radiologist agreement with senior neuroradiologist (κ: 0.52 → 0.58, p < 0.05). Readers described the tool as useful “attention focuses.”	Not clinically deployed.	Voxel-wise color-coded Modic map output visualizing heterogeneous and transitional MC pathology. Rule-based T1/T2 z-score classification system is interpretable and follows the original Modic criteria. Intermediary outputs (vertebral body segmentation, binary MC mask) shown to users to build confidence.	Barriers reported: Small sample size with poor Modic type 3 representation, single institution without multi-institutional validation, and potential annotator bias as training data labelers also participated in the AI-assisted experiment. Facilitators reported: Novel voxel-wise Modic mapping enabling visualization of heterogeneous and transitional pathology; AI assistance significantly improved junior–senior radiologist agreement; model trained on non-standardized multi-parameter clinical data improving real-world applicability.
Georgiev et al. 2023 [40]	CoLumbo standalone: sensitivity 92.70% ± 4.36% (127/137 true positives), specificity 99.04% ± 0.47% (1644/1660 true negatives), PPV 88.81% ± 5.31%, NPV 99.40% ± 0.42%. Overall inter-rater reliability (kappa): radiologist using CoLumbo 92.9%; CoLumbo standalone 89.9%; radiologist without CoLumbo 73%. No AUC reported.	Kappa agreement with majority opinion: CoLumbo-assisted radiologist 92.9% (almost perfect); CoLumbo alone 89.9% (almost perfect); unassisted radiologist 73% (substantial). Of 156 disagreement cases, arbiter agreed with CoLumbo-assisted radiologist in 138 cases (88.5%) and with unassisted radiologist in only 18 cases (11.5%). Prior literature reported human inter-observer kappa of 0.26–0.65 for LSS grading.	CoLumbo standalone: 10/137 false negatives (7.3%) and 16/1660 false positives (1%). Key error categories: borderline dural sac area cases near 100 mm² threshold; stenosis at vertebral body level; naturally reduced sac (e.g., at L4/L5 or sacral level); stenosis from epidural lipomatosis at sacral level not detected.	Prospective multicenter design with consecutive patient enrolment across 3 centres; no separate held-out test set described.	AI-assisted radiologists outperformed both unassisted radiologists and the AI standalone.	Deployed in a real prospective clinical setting across three centres in Bulgaria.	Binary presence/absence output per lumbar level with quantitative dural sac cross-sectional area measurement.	Barriers reported: MRI-only ground truth without CT or electrophysiological validation, data from different MRI systems across sites as a potential source of discrepancy, and performance limitations for borderline dural sac cases and anatomically variable stenosis locations (sacral level, vertebral body level). Facilitators reported: Prospective multicenter consecutive design reducing selection bias; AI-assisted radiologists achieved higher agreement than unassisted radiologists and the algorithm alone.
Hartley et al. 2024 [75]	Best model (optimal angle features only): accuracy 93.98%, sensitivity 96.49% (MI detection), specificity 88.46% (MCI detection), F1 0.957 (p < 0.001). All angle features: accuracy 87.95%, sensitivity 92.98%, specificity 76.92%, F1 0.914. PROMs alone: accuracy 68.67%, sensitivity 91.23%, specificity 18.52%, F1 0.800. Pose estimation criterion validity: MSE 0.35 degrees vs. 3D motion capture.	Not reported for the AI model itself. Expert clinician inter-examiner agreement cited from prior literature: kappa 0.90 (>100 hours training) to kappa 0.66 (<100 hours training); video-based classification kappa 0.55–0.71. BACK-to-MOVE accuracy of 93.98% compared favourably against expert consensus (kappa 0.82, 86% agreement). No kappa reported for the AI model in this study.	Not reported as explicit counts.	Internal validation only; 5-fold cross-validation on 83 participants. Pose estimation validated against synchronized 3D motion capture in a subset of 62 participants.	Not formally evaluated in a clinical deployment study. Model accuracy exceeded inter-examiner agreement of less-experienced clinicians (<100 hours training, kappa 0.66) and video-based expert classification (kappa 0.55–0.71).	Not clinically deployed.	Movement features are clinician-derived and clinically interpretable (range, speed, depth variance, movement stability of spine flexion). Facial blurring applied for patient anonymity. No visual explainability tools (e.g., Grad-CAM) reported..	Barriers reported: Single movement task (spine flexion only) limiting directional subclassification, small dataset from a single centre, video quality sensitivity to lighting and background, and participants wearing sports clothing potentially aiding pose estimation in ways not reproducible in standard clinical settings. Facilitators reported: First automated NSLBP clinical classification using standard video; accuracy comparable to expert consensus without specialized hardware.
Jans et al. 2021 [39]	sCT vs. T1-weighted MRI (CT as reference): Erosion — accuracy 94% vs 86% (p = 0.003), specificity 96% vs 89% (p = 0.01), sensitivity 78% vs 69% (p = 0.49). Sclerosis — accuracy 97% vs 81% (p < 0.001), sensitivity 94% vs 20% (p < 0.001). Ankylosis — accuracy 92% vs 84% (p = 0.04), sensitivity 93% vs 70% (p = 0.001). No AUC reported.	Intrareader kappa: T1-weighted MRI 0.68–0.76; sCT 0.70–0.88; CT 0.77–0.90. Interreader kappa: T1-weighted MRI 0.58–0.74; sCT 0.70–0.90; CT 0.75–0.84. sCT inter- and intrareader reliability was comparable to conventional CT and superior to T1-weighted MRI.	None reported	Quadrant-level diagnostic accuracy analysis (240 quadrants for erosion/sclerosis; 120 half-joints for ankylosis). Intrareader reliability tested in 15 participants by reader 1 at 4-month interval.	sCT improved reader diagnostic confidence for erosion and ankylosis detection compared to T1-weighted MRI. Inter- and intra-reader reliability was comparable to CT.	Commercially available software (BoneMRI v1.1) integrated with hospital PACS via DICOM; automatically receives source MRI, reconstructs sCT, and returns images within 30 minutes with no manual input. Tested in a real prospective clinical setting alongside routine MRI. Not yet standard of care but positioned as an add-on to existing MRI workflow.	sCT output expressed in Hounsfield units, displayed and scored identically to conventional CT in semicoronal reconstructions (1 mm section thickness). Diagnostic confidence rated on a 4-point scale. Fully automatic post-processing with no user interaction required. Output visually interpretable by radiologists using standard DICOM viewers.	Barriers reported: Small sample size (n = 30) limiting patient-level analysis; single tertiary center introducing potential selection bias; no healthy control group; diagnostic accuracy dependent on disease prevalence of the study population. Facilitators reported: Fully automated PACS-integrated pipeline requiring no manual input; sCT reconstruction <30 minutes; superior diagnostic accuracy and reliability vs. T1-weighted MRI for all three structural lesion types.
Ke et al. 2024(65)	AI: sensitivity 97.98%, specificity 98.45%, accuracy 98.21%; mAP 90.08%. Surgeons: accuracy 84.14–92.58%. AI superior to all surgeons (p < 0.05). No AUC reported.	Inter-rater reliability	AI misclassification ~1.79%; surgeons ~7.42–15.86%. Spondylolisthesis had lowest per-class accuracy (84.67%) due to limited training data.	Single-center retrospective; 8:2 training/testing split; 5-fold cross-validation. No external validation or prospective trial.	AI outperformed all surgeons and was ~ 6 × faster (14.5s vs. 87–92.5s).	Deployed at Guilin People’s Hospital for clinical practice and medical education. PACS-integrated DICOM-to-JPEG pipeline. Not externally validated or standard of care.	Color-coded bounding boxes with confidence scores overlaid on MRI. Binary detection only; no severity grading. Designed to complement physician judgement.	Barriers reported: Small testing set (n = 50); single-center retrospective; no external validation; binary output only. Facilitators reported: High accuracy and speed exceeding all surgeons; PACS-integrated workflow; clinically deployed with positive feedback; transfer learning mitigates limited sample size; potential to reduce radiation exposure.
Krabbe et al. 2024 [43]	No formal accuracy metrics (no reference standard). T1w-GRE detected more erosions than T1w-TSE (57 vs 43 quadrants, experts; p = 0.02) and more sclerosis (26 vs 17; p = 0.04). sCT detected more sclerosis (34 vs 17; p < 0.001) and ankylosis (20 vs 13 joint halves; p = 0.02) than T1w-TSE. No AUC reported.	Interreader kappa (experts/non-experts): Erosion — TSE 0.43/0.21, GRE 0.56/0.42, sCT 0.41/0.39. Sclerosis — TSE 0.37/0.30, GRE 0.53/0.39, sCT 0.69/0.40. Ankylosis — TSE 0.83/0.52, GRE 0.88/0.73, sCT 0.64/0.71. sCT is significantly lower than T1w-GRE for ankylosis among experts. No intra-rater data.	None reported	Single-center; n = 19; seven readers assessed all modalities independently in randomized order with ≥10-day washout. No external validation or reference standard.	Both T1w-GRE and sCT improved lesion detection and reader confidence versus T1w-TSE. sCT recommended alongside source T1w-GRE images per BoneMRI Instructions for Use. Performance expected to improve with reader familiarity.	sCT post-processing performed offsite by MRIguidance. Assessed via standard DICOM viewer. Not yet in routine use; positioned as add-on to SIJ MRI protocols for clinical care and trials.	1mm semi-coronal sCT read like conventional CT. Sclerosis conspicuous as bright areas on sCT. Erosion cavities occasionally smoother on sCT than T1w-GRE, suggesting minor spatial information loss. Must be reviewed alongside source T1w-GRE per Instructions for Use.	Barriers reported: Small sample (n = 19); no reference standard; offsite post-processing; reader inexperience with T1w-GRE and sCT; T1w-GRE cannot replace T1w-TSE for fat signal (backfill, fat metaplasia); results may not generalize to higher-resolution T1w-TSE centers or bio-naïve patients. Facilitators reported: Both modalities improved lesion detection and confidence despite reader inexperience; commercially available BoneMRI software.
Lagerstrand et al. 2022 [76]	Fissure model: accuracy 97%, precision 99% (mean of 10 repetitions); strong ROC AUC. Pain model: accuracy 69%, precision 71%; moderate ROC AUC. No sensitivity/specificity reported explicitly.	CT-discogram: intra-rater 96% (axial and sagittal); inter-rater 100%. MRI HIZ assessment: kappa >0.80. IVD segmentation ICC 0.79–0.99 across sub-regions (previously reported).	Fissure model ~3%. Pain model ~31%; recall for pain-negative IVDs only 0.39, indicating frequent false negatives.	Single-center; retrospective; 75/25 split repeated 10 times. Learning curves showed no overfitting. No external validation cohort.	Fissure model could enable non-invasive objective IVD phenotyping, potentially replacing CT-discography.	In-house MATLAB-based software for MRI marker extraction. Not commercially available; not clinically deployed. Research proof-of-concept only.	Binary output (fissure/no fissure; pain-positive/negative) with feature importance scores. No visual output overlaid on images. Requires manual IVD segmentation as input step.	Barriers reported: Small sample (n = 30; 83 IVDs); no external validation; pain reference (PCD) invasive and controversial; PCD may induce false-positive pain responses in adjacent discs; selection bias (LBP patients with discography data only); manual segmentation required; pain model clinically insufficient. Facilitators reported: Unique dataset combining MRI and CT-discograms enabling robust fissure reference standard; fissure model high accuracy with rapid learning curve convergence suggesting low data requirements; objective MRI markers reduce subjective human interpretation.
Lee et al. 2021 [49]	Slice-level (ROI patch + median filter): accuracy 93.80%, recall 93.35%, precision 94.70%, specificity 94.24%, AUC 0.97–0.99 across folds. Subject-level: accuracy 96.06%, recall 100%, precision 94.84%, specificity 86.43%, F1 97.32%. No comparison to clinician performance reported.	Not formally reported for AI model. Ground truth established by consensus of at least two members from a panel of two rheumatologists and one radiologist. Prior literature cited: specialist concordance for sacroiliitis 0.68–0.73.	Not reported as explicit counts.	Single-centre; 5-fold cross-validation with 70/30 subject-level train/test split repeated five times. No external validation cohort.	Positioned as auxiliary diagnostic tool given moderate specialist concordance (0.68–0.73). No formal clinician impact study conducted.	Not clinically deployed. Requires manual ROI annotation by a radiologist as input.	Grad-CAM maps confirm model activation aligns with bone marrow edema lesion areas in most cases. Binary per-slice output aggregated to subject-level diagnosis. Errors identified where sacral bone signal interfered with lesion detection on Grad-CAM.	Barriers reported: Small dataset (n = 79; 815 slices); single-centre; no external validation; manual ROI annotation required limiting full automation; gadolinium contrast required; only bone marrow edema assessed (synovitis, enthesitis, erosion, ankylosis excluded); model not validated against clinician performance. Facilitators reported: Transfer learning enables effective training on small datasets; ROI patch approach improves accuracy by ~10% over whole-image input.
Lee et al. 2023 [48]	Test set: accuracy 94.53–98.44% across grades; sensitivity 100% (Grade 3, Normal), specificity 100% (Grade 4); PPV 82.61% (Grade 1) to 100% (Grade 4); NPV 100% (Grade 3, Normal); F1 64.41% (Grade 1) to 95.38% (Grade 3). No AUC reported. Performance described as at least comparable to two human expert readers.	Not formally reported for AI. Ground truth established by consensus of two musculoskeletal radiologists (9 years’ experience each); CT used as reference standard. Prior literature cited: erosions showed lowest inter-reader agreement (25%) on plain radiographs.	Not reported as explicit counts. Grade 1 had lowest F1 (64.41%) and PPV (82.61%), indicating highest misclassification for early/subtle disease. Grad-CAM examples show cases where model activated incorrect regions despite correct diagnosis, and cases of incorrect diagnosis.	61/19.5/19.5% train/validation/test split (n = 492). No external validation; no cross-validation. Only right SIJ is used, limiting generalizability.	No formal clinician impact or workflow integration study conducted.	Not clinically deployed beyond study context.	The Grad-CAM maps provided for visual validation; confirmed model can localize SIJ despite unsupervised learning.	Barriers reported: Single-center; no external validation; small dataset (n = 492); unsupervised learning limits interpretability and may produce clinically non-meaningful associations; Grade 1 (early sacroiliitis) poorly detected. Facilitators reported: No-code cloud-based platform lowers technical barrier for clinician-researchers; no complex preprocessing or coding required.
Lehnen et al. 2021 [73]	Vertebra/disc labelling: 100%. Disc herniation: accuracy 86.8%, sensitivity 75.3%, specificity 87.9%, PPV 37.2%, NPV 97.4%. Extrusion: accuracy 86.5%, sensitivity 89.1%, PPV 26.3%. Disc bulging: accuracy 75.6%, sensitivity 51.9%. Spinal canal stenosis: accuracy 98.1%, sensitivity 77.1%, specificity 99.0%. Nerve root compression: accuracy 90.5%, sensitivity 71.2%. Spondylolisthesis: accuracy 87.6%, PPV 13.1%. No AUC reported.	Not formally reported for AI. Single expert radiologist (5 years’ experience) served as reference standard; no inter-rater reliability calculated.	Disc herniation: 98 FP, 19 FN. Disc bulging: 153 FP, 64 FN. Spondylolisthesis: 106 FP, 4 FN (PPV 13.1%, driven by algorithm classifying grade 0–1 borderline cases). Nerve root compression: 81 FP, 17 FN. Intraforaminal and small extrusions were missed; herniated disc material misidentified as nerve root in one illustrated case.	Single-center external validation of a commercially developed CNN trained on a separate multicenter dataset. Retrospective; n = 146 consecutive patients. No cross-validation; reference standard from single expert reader only.	High NPVs suggest potential to rule out pathology and reduce radiologist workload for normal studies. Low PPVs (particularly spondylolisthesis 13.1%, disc herniation 37.2%) limit standalone clinical use. Authors propose CNN as supplementary tool rather than replacement; note potential for standardizing reporting and reducing inter-rater disagreement.	CNN processes DICOM files directly and generates an automated written report via software user interface. Mean processing time ~9.5 minutes (CPU-based preclinical version).	Color-coded segmentation overlay on MRI with quantitative measurements (e.g., herniation diameter, dural sac area) and automated text report. Intuitive visual interface.	Barriers reported: Single expert reference standard; single-center retrospective design; small sample (n = 146); exclusion of patients >70 years, post-surgical, or with fractures limits generalizability. Facilitators reported: First external validation of a comprehensive multi-pathology lumbar spine CNN; processed images from two field strengths (1.5T and 3T) with no significant performance difference
Liawrungrueang et al. 2024 [51]	Overall (training/testing/external validation): accuracy 97%/95%/92%; sensitivity 100%/100%/98%; specificity 95.4%/94%/94%; F1 97.77%/95%/95%; ROC-AUC 97%/92%/95%. Per-grade AUC ranged 96.0–97.5%. Prediction error 2.0–2.5% across sets.	Not formally reported.	Prediction error 2.3% (training), 2.0% (testing), 2.5% (external validation).	Three-dataset design: training (n = 1,000), internal testing (n = 500), and external validation (n = 500) from a separate public dataset (SPIDER), with no overlapping images. Balanced class distribution (200/100 per grade per set). No cross-validation or prospective clinical validation.	Positioned as decision-support tool for radiologists to enhance Pfirrmann grading accuracy and reduce diagnostic variability.	Not clinically deployed.	5-class graded output with ROC curves and heatmap visualizations across grades. Bounding box detection localizes affected disc levels. No Grad-CAM or formal explainability tool reported.	Barriers reported: Dataset from symptomatic LBP population only, limiting generalizability; anonymous imaging devices and patient demographics preclude subgroup analysis; Pfirrmann grading subjectivity may affect ground truth quality. Facilitators reported: High accuracy across all Pfirrmann grades including external validation; YOLO architecture enables real-time single-pass detection.
Lim et al. 2022 [52]	Greatest improvement: four-class neural foraminal stenosis — general radiologists κ 0.71 vs 0.39 (p < 0.001); in-training radiologists κ 0.70 vs 0.39 (p < 0.001). Two-class AUC favoured DL-assisted across all subgroups and regions.	Gwet κ reported for all radiologist subgroups with and without DL across three regions (spinal canal, lateral recesses, neural foramina) using four- and two-class grading. Reference standard: single external musculoskeletal radiologist (32 years’ experience).	Not reported as explicit misclassification counts for the radiologist-assisted analysis.	Retrospective crossover study; single-center internal test set (n = 25 patients; 444 images). Eight radiologists assessed studies with and without DL assistance in counterbalanced order with 1-month washout.	Reporting time reduced by 62–74% across all radiologist subgroups (MSK: 124 → 47s; general: 226 → 70s; in-training: 274 → 71s; all p < 0.001).	Publicly available model (GitHub). Tested in supervised reading room setting; not yet prospectively deployed in routine clinical use.	Radiologists can accept or modify predictions; automated report generated detailing level and grade. No Grad-CAM or formal explainability tool. Radiologist remains in control throughout, which authors note aligns with patient preference for human oversight.	Barriers reported: Single-center internal test set (n = 25); no external validation; stenosis grading only — other pathologies (e.g., fractures) not assessed; multiple grading scales in clinical practice may limit generalizability. Facilitators reported: Marked reduction in reporting time across all experience levels; DL equalized performance of general and in-training radiologists with musculoskeletal subspecialists.
Lin et al. 2022 [41]	Original: sensitivity 0.86, specificity 0.92, AUC-ROC 0.92, dice 0.48. Fake-color: sensitivity 0.90, specificity 0.93, AUC-ROC 0.96, dice 0.51. At SI joint MRI level, fake-color outperformed rheumatologist (sensitivity 0.53, specificity 0.67) and matched radiologist (sensitivity 0.94, specificity 0.89).	Interobserver ICC 0.88 (almost perfect) for SPARCC scoring; kappa 0.63 (substantial) for binary sacroiliitis identification. No intra-rater reported.	Original: 2 FP, 2 FN at SI joint level. Fake-color: 1 FP, 1 FN. Low lesion-level PPV (fake-color 0.50; original 0.70) indicating overestimation at lesion level.	Single-center; ~ 8:1:1 train/validation/test split; 5-cycle validation; no external validation.	Fake-color algorithm matched radiologist and outperformed rheumatologist; proposed for screening and disease activity monitoring in settings lacking specialist expertise.	Prototype only; not clinically deployed.	Pixel-wise mask overlay on STIR MRI; interpretable but imprecise at lesion level (dice 0.48–0.51).	Barriers reported: two-investigator ground truth (bias risk); low lesion-level PPV/dice; single-center; no external validation. Facilitators reported: large multi-hospital cohort (389 participants, 5369 images); fake-color mimics real-life slice-comparison; applicable in low-resource settings.
Lin et al. 2024 [42]	Pretrained model: sensitivity 0.90, specificity 0.93 (image); 0.94/0.95 (patient). BME model: sensitivity 0.83, PPV 0.55. Deep lesion: sensitivity 0.86, specificity 0.74; intense lesion: 0.79/0.81. SI joint accuracy 0.90; vessel accuracy 0.88. Dice: sacrum 0.82, ilium 0.80. AI vs. human readers: ICC 0.83, Pearson 0.86.	Interobserver ICC among three readers 0.92. Interobserver ICC (5-day interval) 0.90.	BME model low PPV (0.55) risks SPARCC overestimation. Pretrained model: 5 FN, 50 FP in testing (minimal impact on consecutive-slice scoring). SI joint misidentification predominantly on lateral slices (73% of errors).	Prospective single-center; 317/35/37 train/validation/test split; 10-fold cross-validation; no external validation.	Scoring time ~30 seconds vs. 5–6 minutes manually; more objective intensity thresholding than visual approximation; high agreement with human readers.	Prototype only; end-to-end automated SPARCC scoring pipeline intended for clinical and research use, particularly in low-resource settings.	Outputs numerical SPARCC score with segmentation overlays. Low resolution precluded centimeter depth measurement; deep lesions approximated as >50% SI joint depth.	Barriers reported: small cohort; low BME model PPV; lateral slice SI joint misidentification; Frangi filter cannot isolate presacral vein; low resolution limits depth measurement; no external validation. Facilitators reported: ICC 0.83 with human readers; ~ 30-second scoring; objective vessel thresholding reduces interreader variability; 10-fold cross-validation; applicable in resource-limited settings.
Liu et al. 2024 [68]	ResNet-34 (test set): AUC 0.96, accuracy 91.67%, sensitivity 90.00%, specificity 92.50%, PPV 85.71%, NPV 94.87%. ResNet-34 (external validation): AUC 0.88, accuracy 88.76%, sensitivity 87.36%, specificity 86.74%. Human identification (test): AUC 0.65, accuracy 70.87%. ResNet-34 outperformed DenseNet-121 (AUC 0.87/0.66) and MobileViT_s (AUC 0.82/0.70) on both datasets.	Not formally reported.	ResNet-34 test set: 4 FN, 6 FP (out of 120). External validation: 193 FP, 61 FN (out of 611). Human identification is substantially worse on external validation (AUC 0.41).	Multicenter retrospective; 3 hospitals; 493 training/ 120 test/ 611 external validation (PSM-matched); 8:2 train/validation split within development set; no cross-validation reported.	ResNet-34 substantially outperformed human readers (AUC 0.96 vs. 0.65); proposed as rapid auxiliary diagnostic tool to guide surgical planning without CT radiation exposure.	Proposed as pre-operative decision-support tool using MRI alone, reducing reliance on CT; not yet clinically deployed.	Binary classification output; no explainability tools (e.g., Grad-CAM) reported. ROC curves and confusion matrices provided for model comparison.	Barriers reported: limited sample size (n = 1,224); only one external validation site; variable disease severity across patients; MRI less sensitive than CT for calcification detection; no interpretability analysis. Facilitators reported: multicenter design; PSM-matched external validation; outperformed human readers and two comparator models; MRI avoids ionizing radiation; fast inference suitable for clinical workflow.
Liu et al. 2024 [67]	PRNet (best): accuracy 87.3%, sensitivity 82.1%, specificity 94.3%, PPV 95.0%, F1 88.1%. Baseline CNN: accuracy 77.5%. Comparator radiologists (3, > 10 years): accuracy 83.3–94.9%, sensitivity 94.9–97.4%, specificity 78.6–89.7%. PRNet outperformed all comparator DL models (ResNet18, VGG16, GFNet, Swin UNETR, etc.). 5-fold cross-validation mean accuracy 84.9% (SD 0.016).	Three radiologists evaluated independently; accuracy ranged 83.3–94.9% across readers, highlighting inter-reader variability. No ICC or kappa reported.	Not explicitly reported as FP/FN counts. Radiomics + L1 regularization reduced false positives; without L1: accuracy 86.3%, SPE 85.1%; with L1: accuracy 87.3%, SPE 94.3%, PPV 95.0%.	Single-center, private dataset; 952 patients (1,904 SIJ data blocks); 8:1:1 train/validation/test split; 5-fold cross-validation. No external validation.	Performance comparable to radiologists with 10 years’ experience; Grad-CAM activation maps align with radiologist diagnostic patterns for osteosclerosis and joint fusion; proposed as CAD tool to reduce subjective inter-reader variability.	Not clinically deployed	Grad-CAM visualizations provided, showing frequency-domain feature activation aligned with lesion sites (osteosclerosis, joint fusion). Radiomics features add interpretable prior knowledge.	Barriers reported: single-centre dataset with potential regional bias; coarse SIJ segmentation (DSC 68.8%) introduces ROI noise; imbalanced grade IV samples; label noise from subjective grading standards; no external validation; transformer-based frequency extraction not yet implemented. Facilitators reported: novel OE-FFT/AP-FFT modules improve low-frequency feature extraction; radiomics reduces false positives; multi-task learning with 5-class labels improves binary classification; Grad-CAM enhances clinical interpretability; performance matches experienced radiologists.
Liu et al. 2024 [66]	Internal test set: accuracy 86.25%, recall 87.77%, precision 84.92%, F1 85.60%. SSD recall: 92.86% (low signal), 88.64% (high signal). External test set: accuracy 75%, recall 77.08%, precision 77.80%, F1 74.97%. SSD recall: 71.74%/73.91%. No AUC reported.	Inter-observer kappa (Physician 1 vs. Physician 2): 0.768 internal (substantial), 0.681 external (substantial). Observer-classifier kappa: 0.717 internal (substantial), 0.519 external (moderate). No intra-rater reliability reported.	Internal: 26/35 low signals correct (9 FN); 43/45 high signals correct (2 FP). External: 22/24 low signals correct (2 FN); 20/32 high signals correct (12 FN). Notable performance drop on external dataset.	Single-centre retrospective; internal dataset n = 140 (725 augmented images), 8:1:1 train/validation/test split; external validation n = 28 from different MRI equipment (same institution); no multi-institution external validation.	Model achieved substantial agreement with experienced physicians (kappa 0.717 internal).	Not clinically deployed	Outputs MC type classification with bounding box localization; confusion matrices provided. No explainability tools (e.g., Grad-CAM) reported. Binary signal labeling system (high/low) is straightforward but limits nuanced interpretation.	Barriers reported: small dataset (n = 140 internal; augmented to 725); single annotator for ground truth introduces subjectivity; single-institution data limits generalizability; notable accuracy drop on external set (86% → 75%); no multi-institution validation. Facilitators reported: two-network approach resolves noise issues vs. single-network; SSD localization reduces edge noise before classification; substantial observer-classifier agreement achieved; transfer learning from ImageNet improves efficiency with limited data.
Miyo et al. 2023 [77]	No standalone diagnostic accuracy metrics (sensitivity/specificity/AUC) reported. DLR quantitative image noise significantly lower than hybrid IR (axial: 14.8 vs. 21.4; sagittal: 14.2 vs. 20.6; P < .0001). DLR rated significantly better for subjective noise, structural depiction, and overall image quality by both readers (P < .006). LSS grading distributions similar across reconstruction methods.	Interobserver agreement for LSS grading: hybrid IR kappa 0.732 (95% CI 0.712–0.751; good); DLR kappa 0.794 (95% CI 0.781–0.807; good); DLR significantly better than hybrid IR. No intra-rater reliability reported.	Not applicable	Single-center retrospective; n = 30 consecutive patients; within-patient comparison (same scan reconstructed with both algorithms); two blinded readers; no external validation.	DLR improved interobserver agreement in LSS grading vs. hybrid IR; authors propose DLR-enhanced CT as potential alternative to MRI for LSS evaluation in patients with MRI contraindications or limited MRI access.	DLR is a reconstruction algorithm applied at CT post-processing stage; compatible with existing PACS workflow; enables retrospective LSS evaluation from prior CT scans without additional imaging.	DLR produces standard CT images; output is directly interpretable by radiologists with no additional interface required. Improved structural depiction of intervertebral disc, ligamentum flavum, and sacroiliac joint compared to hybrid IR.	Barriers reported: small sample (n = 30); no MRI comparison to validate CT-based LSS grading; no orthopedic clinical correlation with symptoms; postoperative/metallic implant cases excluded; correlation between CT and MRI findings unestablished. Facilitators reported: within-patient design controls for confounding; significant noise reduction and image quality improvement; improved interobserver agreement; applicable to patients with MRI contraindications; compatible with retrospective review of existing CT scans.
Nigru et al. 2024 [72]	Selected results: disc narrowing accuracy 86.7%, kappa 0.799; central canal stenosis accuracy 97.1%, kappa 0.749; spondylolisthesis accuracy 98.3%, kappa 0.705; endplate defects accuracy 94.2–94.8%, kappa 0.732–0.745; marrow changes accuracy 93.1–94.0%, kappa 0.731–0.756; Pfirrmann accuracy 79.6%, kappa 0.738. Weaker performance: foraminal stenosis accuracy ~85%, kappa 0.457–0.473; herniation accuracy 79.0%, kappa 0.546.	Inter-rater reliability between two radiologists (17 and 38 years’ experience) on 60 patients (289 discs): kappa 0.734–0.930 across all features; LCCC 0.797–1.00; MCC 0.695–0.930. High consistency supported single-radiologist annotation for remaining 293 patients. No intra-rater reliability reported.	Notable misclassifications: Pfirrmann grades 1/2 confusion (87 grade-1 cases labelled grade 2; 48 grade-2 labelled grade 1).	Single-center external validation (private Italian clinic); retrospective; n = 353 patients, 1,747 IVDs; AI-assisted annotation protocol (radiologists reviewed SpineNetV2 predictions and corrected discrepancies).	AI-assisted annotation workflow used; authors report efficiency gains from automated predictions reviewed by radiologists. SpineNetV2 performance comparable or superior to prior external validations for most features.	Open-source model applied via publicly available Python code; tested in a real clinical imaging service (10,000 + procedures/year); designed as decision-support tool for disc pathology grading.	Outputs numerical grades per pathology per disc level; confusion matrices provided for all 11 features.	Barriers reported: sagittal-only MRI limits foraminal stenosis and herniation accuracy; spondylolisthesis multi-disc false positives; AI-assisted annotation may inflate agreement; no external validation for some features in prior literature. Facilitators reported: most comprehensive external validation of SpineNetV2 to date (all 11 features); open-source model enables reproducibility; high inter-rater agreement supports annotation quality; strong performance across 9 of 11 pathologies supports clinical utility.
Ono et al. 2023 [53]	Test set (n = 640 LV images): accuracy 0.894, sensitivity 0.836, specificity 0.920, AUC 0.876; fresh-positive sensitivity 0.748, specificity 0.930, AUC 0.834. External validation (n = 662 LV images): accuracy 0.867, sensitivity 0.674, specificity 0.866, AUC 0.768; fresh-positive sensitivity 0.538, specificity 0.925, AUC 0.721. YOLOv5 detection: mAP(0.5) 0.995 validation, 0.982 test.	Interobserver kappa 0.801 (almost perfect). Intraobserver kappa: Rater 1 = 0.821, Rater 2 = 0.861 (both almost perfect). Consensus required in 141/523 cases.	Test set: FP rate 0.161, FN rate 0.077. External validation: FP rate 0.288, FN rate 0.111. Notable fresh OLVF confusion with old OLVF in external validation (confusion matrix: 29 fresh predicted normal, 13 predicted old, 49 correct).	Two-institution retrospective; Institution 1 (n = 415 patients, 3,481 LV images) for training/validation/test (8:2 split then ~1/10 held for test); Institution 2 (n = 137 patients, 662 LV images) for external validation; 5-fold cross-validation for CNN training; STARD 2015 guidelines followed.	Fresh OLVF sensitivity (74.8% test; 53.8% external) substantially exceeds prior radiograph-only human sensitivity (~52%); proposed to reduce unnecessary MRI by improving radiograph-based triage; eliminates need for manual vertebra selection; functions independently of physician experience.	Not tested; potential discussed	Outputs 3-class probability scores per vertebra; no explainability tools (e.g., Grad-CAM) reported.	Barriers reported: excludes severe crush, scoliosis, and metallic implants; limited generalizability to pathological fractures; relatively small sample; no explainability; manual image transfer required for inference; sensitivity drop for fresh OLVF in external validation. Facilitators reported: first 3-class (normal/old/fresh) OLVF classifier on radiographs; fully automated vertebra detection eliminates manual ROI; multi-institution external validation; performance exceeds human radiograph-alone sensitivity; applicable at any radiography-equipped facility.
Redeker et al. 2024 [62]	Main model (test set, n = 209): accuracy 0.9234, sensitivity 0.9586, specificity 0.8438, F1 0.9456, AUC-ROC 0.9717. Sensitivity analysis (imputed full dataset, n = 282 test): accuracy 0.8936, sensitivity 0.9545, specificity 0.7500, AUC 0.9653. Comparator ASAS algorithm (test set): accuracy 0.8086, sensitivity 0.8276, specificity 0.7656, F1 0.8571.	Not formally reported for the AI model.	Test set: 6 axSpA misclassified as non-axSpA (FN); 10 non-axSpA misclassified as axSpA (FP) out of 209 patients. Misclassified patients showed clinical profiles resembling the opposite group.	Single-center retrospective; 70:30 stratified train/test split (492 training, 209 test); sensitivity analysis with multiple imputation on full n = 939 dataset; two-step sub analyses (clinical-only first, then MRI); no external validation.	Outperformed ASAS algorithm; two-step approach could reduce MRI need in ~50% of patients; model applicable without HLA-B27 in sub analysis (accuracy 93.3%), relevant in low-HLA-B27 prevalence populations; proposed to reduce mean 6.8-year diagnostic delay.	Not implemented; discussed	Feature importance assessed via mean decrease accuracy (MDA); top contributors: HLA-B27 (0.055), insidious LBP onset (0.038), SIJ erosion on MRI (0.037), elevated CRP (0.018).	Barriers reported: single-center tertiary setting with high axSpA pretest probability limits generalizability to primary/secondary care; imbalanced dataset (70% axSpA); no external validation yet. Facilitators reported: high AUC (0.97) outperforming ASAS algorithm; two-step approach reduces MRI burden; HLA-B27-free sub analysis extends applicability.
Roels et al. 2023 [57]	Quadrant-level, cross-validation (T2 only, no masking): AUC 94.5%, B-ACC 80.5%, F1 64.1%, PPV 65.3%, recall 63.4%, FPR 2.4%. Quadrant-level, TEST set: AUC 88.2%, B-ACC 72.1%, F1 50.8%, PPV 55.0%, recall 47.2%, FPR 3.1%. Patient-level: B-ACC 81.6% (cross-validation), 81.4% (TEST); recall 90% at precision ~70–75% (TEST). Deep inflammation (TEST): AUC 91.1%, F1 44.4%.	Reader ICC for ground truth annotation: 0.77–0.90 (across prior studies). No kappa or ICC reported for the AI model itself.	Not reported as absolute counts. Substantially reduced recall and precision in healthy controls (recall 40.3%, PPV 15.5%) versus SpA cohort (recall 68.5%, PPV 66.3%).	Single-centre (Ghent University Hospital); three prospective cohorts (BEGIANT, POPAS, HEALTHY) used for training/cross-validation (5-fold); independent SpA-only TEST set (n = 243 scans, 188 patients); no external multicentre validation. Ablation study comparing input modalities and masking configurations.	First fully automated quadrant-level BME prediction pipeline, removing need for manual ROI selection. Tunable sensitivity/specificity threshold: high-sensitivity mode (B-ACC 87.5%, recall 85.5%) for screening; high-specificity mode (B-ACC 72.0%, PPV 76.4%) for research. Patient-level alert function (90% recall, > 70% precision) could flag high-risk cases for expert review.	Not yet deployed clinically; proof-of-concept only. SPARCC score automation not yet feasible.	Integrated gradient (IG) method used to generate attention maps identifying inflammation-relevant pixels; confirmed model attends to biologically plausible regions. IG selected over CAM/Grad-CAM for theoretical guarantees and computational efficiency. Output is quadrant-level probability enabling semiquantitative assessment; supports longitudinal monitoring potential.	Barriers: Single-center data with largely uniform MRI acquisition; TEST set SpA-only (no controls), limiting diagnostic applicability; SPARCC automation not yet reliable; no external validation. Facilitators: First fully automated quadrant-level pipeline; large real-world annotated dataset across three cohorts; interpretable attention maps; code publicly available (GitHub).
Seo et al. 2023 [78]	No sensitivity/specificity/AUC reported. Intermethod agreement (DL-Dixon vs. routine Dixon) was almost perfect for disc herniation, facet arthropathy, uncovertebral arthropathy, and central canal stenosis (κ: 0.823–0.980); substantial to almost perfect for foraminal stenosis (κ = 0.705–0.955). Subjective image quality superior for all four anatomical structures in DL-Dixon (p < 0.001–0.002). Scan time reduced by 23.76% (181 → 138s). NU slightly higher in DL-Dixon (17.07 vs. 15.88, p = 0.015).	Interreader agreement reported per sequence. Routine Dixon: moderate for foraminal stenosis (κ = 0.596), substantial for facet and uncovertebral arthropathy, almost perfect for disc herniation and central stenosis. DL-Dixon: improved foraminal stenosis agreement from moderate to substantial (κ = 0.760); otherwise comparable. No intra-rater reliability reported.	Not reported.	Retrospective single-center (n = 50); both sequences acquired in the same patients (within-subject comparison); two independent radiologists (19 years and 3 years’ experience); images presented in randomised order. No external validation; small sample.	23.76% scan time reduction without significant loss of lesion detectability. Improved interreader agreement for foraminal stenosis. Superior structural visibility may aid detection of subtle pathology. Motion artifact amplification in a subset is a potential diagnostic concern.	No workflow changes required beyond sequence substitution.	Not interpretable directly; black-box reconstruction; validated statistically	Barriers: Small cohort (n = 50); only 2 readers; characteristic DLR image texture may have enabled readers to distinguish sequences (detection bias); matrix size difference confounds image quality comparison; no external validation. Facilitators: Within-subject design eliminates patient variability; almost perfect intermethod agreement across most lesion types supports diagnostic equivalence; seamless integration into existing protocol.
Shahzadi et al. 2023 [50]	Best results with 12.5k augmented dataset: multi-ROI accuracy 97.01%, precision/recall/F1 macro avg. 0.97; single-ROI accuracy 97.71%, precision/recall/F1 macro avg. 0.98. Without augmentation (n = 1,545 original images): multi-ROI 36.47%, single-ROI 35.66%.	Not reported for the AI model. Ground truth derived from expert radiologist grading of a pre-annotated public dataset; no inter-rater agreement statistics provided.	Not reported as absolute counts. Lowest per-class performance in multi-ROI: mild class (precision 0.94, recall 0.92, F1 0.93). With 5k data, moderate class precision dropped to 0.74. No confusion matrix presented.	Publicly available dataset (515 patients, 1,545 axial MRI images); 80:20 train/test split (randomly selected); 5-fold cross-validation; three augmented dataset sizes (5k, 10k, 12.5k) compared.	Proposed as CAD tool to assist decision-making in LSS grading, addressing radiologist shortage.	Not deployed; computer-aided detection (CAD) tool proposed	No explainability methods (e.g., Grad-CAM, saliency maps) applied.	Barriers: Publicly available dataset with limited size (1,545 original images) requiring heavy augmentation; model near-random without augmentation (36% accuracy); single-ROI (AAP) focus limits broader anatomical coverage; no interpretability analysis. Facilitators: High accuracy with augmented data (97.71%) outperforms multiple prior CNN baselines.
Soin et al. 2022 [70]	Overall accuracy is approximately 72% compared to practitioner-assigned diagnoses. No sensitivity, specificity, AUC, F1, or per-class metrics reported. No statistical testing was performed; comparison made using simple averages only.	Not reported.	Not reported as absolute counts or per-class rates. Approximately 28% of predictions did not match practitioner diagnosis; no breakdown by diagnosis category provided.	Prospective single-center pilot study (n = 246 consecutive patients); single 80:20-style comparison of algorithm predictions vs. chart diagnoses; no train/test split, cross-validation, or external validation described.	Proposed as an augmented decision-support tool to guide clinicians toward the most likely diagnosis and reduce treatment bias (e.g., specialty-driven over-recommendation).	Not implemented; proof-of-concept	Decision tree architecture is inherently interpretable (rule-based). No feature importance ranking, visualization, or explainability output reported.	Barriers: Single-center pilot with small sample (n = 246); no statistical testing; incomplete data entry by some patients limiting dataset; algorithm trained on practitioner-assigned diagnoses, generalizability to other pain practices unclear. Facilitators: Prospective design; 72% accuracy comparable to published algorithmic diagnostic approaches using diagnostic nerve blocks; low-cost, framed within established ASIPP algorithmic framework for spinal pain management.
Su et al. 2022 [54]	Internal test (n = 2,299 MRIs, 153 patients): LDH accuracy 84.17%, sensitivity 90.7%, specificity 92.2%, F1 84.1%, Gwet k = 0.80; LCCS accuracy 87.0%, sensitivity 65.2%, specificity 94.5%, F1 86.8%, k = 0.85; LNRC accuracy 81.21%, sensitivity 79.2%, specificity 92.9%, F1 80.0%, k = 0.78. Dichotomous AUC (grade 0–1 vs. 2–3): LDH 0.97, LCCS 0.98, LNRC 0.95. External test (n = 1,273 MRIs, 100 patients): LDH 74.16%, k = 0.67; LCCS 79.65%, k = 0.77; LNRC 74.16%, k = 0.69. Dichotomous AUC: LDH 0.95, LCCS 0.98, LNRC 0.87. Grade 2–3 precision markedly lower across both datasets (e.g., LCCS grade 2 precision 27.9% internal, 9.1% external).	Substantial-almost perfect agreement with clinicians: Internal kappa: LDH 0.80, LCCS 0.86, LNRC 0.78; External kappa: LDH 0.67, LCCS 0.77, LNRC 0.69. Human-human kappa higher (LDH 0.93 internal, 0.88 external).	Detailed confusion matrices provided. Worst performance consistently for grades 2–3 due to class imbalance (e.g., LCCS grade 3: only 14 internal test cases; LNRC grade 2 F1 11.2% internal). Grade 0 and 1 classifications were substantially more accurate across all three tasks and both datasets.	Retrospective two-centre study; internal dataset 15,254 MRIs from 1,015 patients (70/15/15 train/validation/test split); external test dataset 1,273 MRIs from 100 patients at a separate institution.	Improved grading efficiency, reduced radiologist workload, high-speed inference (~11 ms per image)	Not yet clinically implemented; proof-of-concept research study	Output is a structured four-grade classification for three diseases simultaneously, directly mapped to established clinical grading systems. Confusion matrices and ROC curves reported.	Barriers: Class imbalance for severe grades (grades 2–3) resulting in low precision/recall for clinically important categories; axial MRI only (no sagittal sequences); bounding box annotation required for training. Facilitators: Large internal dataset (15,254 MRIs); external validation at separate institution; simultaneous multi-task grading mirrors real clinical co-occurrence; comparable or superior LCCS accuracy to prior studies.
Tang et al. 2024 [79]	No sensitivity/specificity/AUC for pathology diagnosis reported. No significant difference in detection rate of any pathology between TSE-DL and TSE-SD (all p ≥ 0.219). TSE-DL SNR significantly higher across all anatomical structures and sequences (all p < 0.001). Overall image quality superior or equivalent on TSE-DL; significant improvement in sagittal T1W (p = 0.003/0.008) and transverse T2W (p = 0.022, reader 1). Diagnostic confidence not significantly different (p ≥ 0.081). Scan time reduced by 45% (317s → 175s).	Interprotocol (intrareader) agreement almost perfect for all pathologies for both readers (κ = 0.84–1.00). Intraprotocol interreader agreement: moderate to almost perfect for TSE-SD (κ = 0.58–1.00); substantial to almost perfect for TSE-DL (κ = 0.61–1.00). Qualitative image quality interreader agreement fair to substantial (weighted κ = 0.26–0.73); poor for artifacts on sagittal T2W (κ = 0.13). SNR interreader ICC moderate to almost perfect (0.54–0.94).	Not reported as absolute misclassification counts. Minor discordances noted: e.g., disc herniation detected by TSE-DL only in 5/45 cases and by TSE-SD only in 1/45; Modic changes detected by TSE-DL only in 3/20 cases.	Prospective single-center study (Shanghai Renji Hospital, n = 31); within-subject design (both sequences in all patients); two radiologists with >10 years’ experience.	45% scan time reduction reduces patient discomfort and motion artifacts, potentially improving diagnostic yield in patients with severe pain.	Proof-of-concept; feasible since SubtleMR is FDA/CE cleared	Output standard MRI images (interpretable); network itself is black box.	Barriers: Small sample (n = 31); single scanner/center; no sample size pre-calculation; no noninferiority testing; fat-suppressed T2W showed increased aliasing artifacts; contrast-enhanced sequences not evaluated. Facilitators: Prospective within-subject design; FDA/CE clearance supports clinical readiness; trained on >1 million image pairs across vendors; 45% scan time reduction clinically meaningful; no significant loss of detection performance across 8 pathology types.
Triantafyllou et al. 2023 [71]	XGBoost (best model): AUC-ROC 0.71 (95% CI 0.58–0.84), confirmed stable on 1000-iteration bootstrap. From confusion matrix (Figure 7): sensitivity ~0.75, specificity ~0.88, PPV ~ 0.45, NPV ~ 0.88, accuracy ~0.71, F1 ~ 0.61 (values read from bar plot). Comparators: Logistic Regression AUC 0.61, SVM 0.61, Random Forest 0.66.	Ground truth established by consensus of two highly experienced musculoskeletal radiologists (40 and 10 years’ experience) blinded to clinical data, applying EULAR criteria. No kappa or ICC reported for radiologist agreement.	15 FN (inflammatory BME missed), 6 FP (non-inflammatory classified as inflammatory) out of 71 test cases. Low PPV (≈0.45) reflects class imbalance (26% positive) and limits standalone diagnostic utility.	Retrospective single-centre study (University Hospital of Heraklion, n = 177 patients, 354 SIJ segments); 80:20 stratified train/test split; bootstrapping with 1,000 resamples for AUC confidence intervals. No external validation; no cross-validation reported.	Not directly measured; potential to aid less experienced radiologists	Not implemented; suggested PACS integration in future	Feature-based radiomics; interpretable features (skewness, wavelet transforms, minimum intensity)	Barriers: Single-center retrospective design; small sample relative to feature space (risk of overfitting); moderate AUC (0.71) insufficient for standalone diagnosis; no external validation; non-contrast MRI may miss enthesitis/synovitis. Facilitators: Largest radiomics dataset for this indication at time of publication; high NPV useful for ruling out inflammatory sacroiliitis.
VanderGraaf et al. 2024 [36]	Multiclass model: weighted κ = 0.86 (95% CI 0.82–0.90) vs. consensus ground truth; R3 κ = 0.85 (0.80–0.89), R4 κ = 0.73 (0.68–0.79). Binary model: AUC 0.98 (0.97–0.99); at Youden’s index — sensitivity 93% (91–96%), specificity 91% (87–95%), accuracy 0.93, PPV 0.84, NPV 0.96. Comparators: R3 sensitivity 74%/specificity 98%; R4 sensitivity 54%/specificity 99%. FSL was the single most influential feature (removal reduced multiclass κ to 0.72, binary AUC to 0.93).	No explicit inter-rater kappa between R1 and R2 prior to consensus reported; no intra-rater reliability reported.	Notable model misclassifications: grade 1 predicted as grade 0 in 65 cases; grade 2 predicted as grade 1 in 77 cases. Binary model: at Youden’s threshold, 7% FN rate and 9% FP rate. R4 showed substantially higher FN rate (sensitivity 54%) than the model.	Retrospective single-center study (Radboud University Medical Center, n = 186 patients, 683 IVD levels); tenfold stratified cross-validation; no external test set. Reported in accordance with CLAIM 2024 checklist. Post-operative and low-quality MRI scans excluded.	Eliminates need for axial sequences for LCCS grading, potentially reducing acquisition time and simplifying protocols. Model sensitivity (93%) substantially exceeds radiologist sensitivity (54–74%) at matched specificity, suggesting improved detection of clinically significant stenosis.	Proof-of-concept; potential PACS integration; outputs traceable and interpretable	Model interpretable (CSA, APD, FSL metrics); more transparent than CNNs	Barriers: Single-center retrospective study; no external validation; small dataset (683 IVD levels); T12-L1 level underrepresented (n = 12); post-operative and implant cases excluded; does not assess lateral recess or foraminal stenosis. Facilitators: Almost perfect multiclass agreement matching senior radiologists; fully interpretable quantitative feature pipeline; novel automated FSL measurement; SPIDER-trained segmentation backbone publicly available; CLAIM 2024-compliant reporting.
Yoo et al. 2023 [55]	Radiologist-average sensitivity/specificity vs. standard images. Central canal stenosis: DL_coarse 0.81/0.88, DL_fine 0.82/0.90, standard 0.83/0.88 (all p>=0.05). Neural foraminal stenosis: DL_coarse 0.75/0.97, DL_fine 0.70/0.97, standard 0.74/0.96 (all p>=0.05). Disc herniation: all arms ~0.76–0.77 sensitivity, ~ 0.93–0.97 specificity (all p>=0.05). Annular fissure: sensitivity higher on both DL arms (p < 0.001), specificity lower (p < 0.001). No AUC reported.	No kappa or ICC reported. Reference standard by consensus of two senior neuroradiologists (20 and 12 years’ experience).	Not reported as counts.	Reconstruction algorithm pre-trained on separate multi-hospital dataset. Subgroup analyses by scanner vendor (Philips Ingenia, Siemens Avanto). No external validation.	Average 32.3% reduction in acquisition time. Diagnostic performance preserved for major targets.	Commercially available post-acquisition software; DICOM-based, vendor-neutral, no k-space access required.	Black-box CNN with CAMs for heatmap interpretability	Barriers: small single-center sample precluded non-inferiority testing; limited scanner types at 1.5T only; facet arthrosis excluded due to ceiling prevalence; incidental findings not evaluated; DL_fine may amplify artefacts; higher false positive rate for subtle findings requires pre-deployment calibration. Facilitators: commercially available, vendor-neutral, no k-space dependency; 32.3% scan time reduction with preserved diagnostic performance for major targets; similar or superior image quality metrics; two selectable outputs; prospective design; multi-hospital training data supports generalizability.
Zhang et al. 2024 [37]	Best single CNN (ResNet50, T1WI), testing: AUC 0.839, accuracy 0.804, sensitivity 0.807, specificity 0.800. Best ensemble (XGBoost), testing: AUC 0.868, accuracy 0.825, sensitivity 0.825, specificity 0.868. Combined model, testing: AUC 0.910, accuracy 0.856, sensitivity 0.877, specificity 0.825, F1 0.877. Combined model outperformed all others (p < 0.05). Good calibration (Hosmer-Lemeshow p > 0.05); DCA favoured combined model.	Not formally reported.	Not reported as counts.	Five-fold cross-validation for XGBoost tuning. No external validation.	Proposed to assist inexperienced radiologists and clinicians with axSpA-related sacroiliitis diagnosis.	Proof-of-concept; designed for PACS integration	CNNs black-box; ensemble and combined models provide probability scores; clinical variables add interpretability	Barriers: retrospective single-centre design; no external validation; limited to oblique coronal orientation; no HLA-B27 or laboratory variables; segmentation accuracy affects downstream diagnosis. Facilitators: fully automated end-to-end pipeline; multimodal fusion outperformed single-modality models; AUC 0.910 with accessible clinical inputs; three-scanner training improves generalizability; Grad-CAM supports interpretability; DCA confirms clinical utility.
Zhang et al. 2023 [58]	3D CNN): validation set micro-average AUC 0.92, accuracy 0.894; class 0/1/2 AUCs 0.91/0.80/0.96. Test set micro-average AUC 0.91, accuracy 0.802; class 0/1/2 AUCs 0.94/0.82/0.93. Class 1 consistently lowest across all metrics. Sacroiliitis diagnosis (combining unilateral grades): validation -- sensitivity 0.917, specificity 0.943, accuracy 0.930; test -- sensitivity 0.913, specificity 0.864, accuracy 0.901. No statistically significant difference between model and radiologists of any seniority for final diagnosis (p > 0.05).	No formal kappa or ICC reported. Ground truth defined as consensus of three veteran musculoskeletal radiologists (20 years’ experience); disagreements resolved by discussion.	Confusion matrices provided. Class 1 had the highest misclassification rate across both sets. Validation set class 1 recall: 0.500; test set class 1 recall: 0.303. Authors attribute this to limited class 1 training data (15% of training set) and inherent diagnostic difficulty of grade II.	Retrospective, two-centre. Internal 80:20 train/validation split (546/142 SIJs, Fifth Affiliated Hospital). External test set from Zhuhai People’s Hospital (182 SIJs). Test set grading distribution significantly different from training/validation sets (p < 0.05). No prospective or further external validation.	Model performance comparable to radiologists of all seniority levels for final sacroiliitis diagnosis. Superior to junior and senior for class 1 on validation set. Fully automated pipeline eliminates manual segmentation.	Proof-of-concept; intended for PACS integration	Segmentation transparent; grading CNN black-box but outputs clinical grade labels	Fa Barriers: retrospective two-centre design; no gold standard for sacroiliitis diagnosis; small dataset, particularly class 1 (15% of training set); model underperforms for class 1; only axial CT orientation supported; expert consensus used as ground truth may itself contain errors. Facilitators: fully automated pipeline requiring no user intervention; external test set included; three-class grading reflects clinical modified New York criteria; nnU-Net self-configures without manual tuning; CT offers highest specificity for structural lesions.
Zhang et al. 2023 [64]	Detection: internal mean IoU 0.82, precision 98.4%, sensitivity 99.4%; external mean IoU 0.70, precision 96.3%, sensitivity 97.8%. Classification: internal test -- overall accuracy 87.70% (95% CI 86.59–88.86%), AUC 0.965 (95% CI 0.962–0.968), ICC 0.87 (95% CI 0.86–0.88); external test -- overall accuracy 74.23% (95% CI 71.83–76.75%), AUC 0.916 (95% CI 0.908–0.925), ICC 0.79 (95% CI 0.76–0.81). Grade 3 had the lowest F1 scores (56.60% internal, 45.45% external). No comparator radiologist performance reported.	ICC reported for model vs. reference standard: internal 0.87 (good), external 0.79 (good). Reference standard established by consensus of multiple radiology experts and senior spinal surgeons per MSU criteria. ROI bounding boxes drawn by one expert spinal surgeon.	Confusion matrices provided. Grade 3 had the lowest sensitivity (50.00% internal, 71.43% external) and precision (65.22% internal, 33.33% external), attributed to class imbalance (Grade 3 = 0.9–2.2% of datasets). Grade 1 also underperformed relative to Grades 0 and 2.	Retrospective, two-centre. Internal 80:20 train/test split (12,012/3,237 images; Fifth Affiliated Hospital, Sun Yat-sen University). External test set from a separate institution (Third Affiliated Hospital of Southern Medical University; 1,273 images, 100 patients). No prospective validation.	Model processes one image in ~20 ms vs. 2.3–6.5 s manually. Accuracy superior to prior multiclass models for related conditions (67.1–86.9%).	Proof-of-concept; suitable for integration into radiology diagnostic pipelines	MSU-based grading interpretable by clinicians; Faster R-CNN bounding boxes for visualization	Barriers: retrospective two-centre design with potential selection bias; class imbalance for Grades 2 and 3 reduces precision; only axial T2W images used; single surgeon drew all bounding boxes. Facilitators: fully automated end-to-end pipeline; external test set included; MSU grading directly links to surgical decision-making; 20 ms inference vs. seconds for manual analysis; model-expert agreement; accuracy superior to prior multiclass LDH models.
Zhang et al. 2024 [38]	Validation cohort (n = 189). Best clinical model (TabNet): accuracy 84.66%, AUC 87.64% (95% CI 0.81–0.93), sensitivity 91.55%, specificity 63.83%, F1 89.97%, MCC 57.58%. Best imaging model (TabNet): accuracy 84.66%, AUC 87.32% (95% CI 0.81–0.92), sensitivity 88.03%, specificity 74.47%, F1 89.61%, MCC 60.49%. CCMRT (TabNet, combined): accuracy 88.36% (95% CI 0.84–0.93), AUC 93.23% (95% CI 0.89–0.97), sensitivity 90.85%, specificity 80.85%, F1 92.14%, MCC 69.82%. CCMRT significantly outperformed best imaging model (p = 0.049). Diagnosis of one patient takes ~0.7 s.	Not formally reported.	Not reported as counts. Average clinical model specificity 52.66% and MCC 40.52% indicate notable misdiagnosis rate. CCMRT improved specificity to 80.85% and MCC to 69.82%, reducing misdiagnosis and missed diagnoses vs. clinical and imaging models alone.	Random 8:2 train/validation split (n = 753/189). No significant difference between cohorts (p > 0.05). No external validation; authors acknowledge this as a limitation.	Diagnosis time ~0.7 s per patient. Feature importance visualization allows clinicians to identify which features drove each prediction. Model not dependent primarily on BME, aligning with individualised precision medicine.	Not yet clinically integrated, proposed support tool	TabNet provides built-in feature importance scores via attentive transformer masks.	Barriers: retrospective single-center design with selection bias; no external validation; manual MRI feature extraction is time-consuming and subjective; BME lacks specificity; combined model outperforms clinical and imaging models alone; TabNet interpretability supports clinician trust; structural lesion inclusion addresses BME non-specificity; fast inference (~0.7 s); accessible input features from routine care.

**Fig 2:**
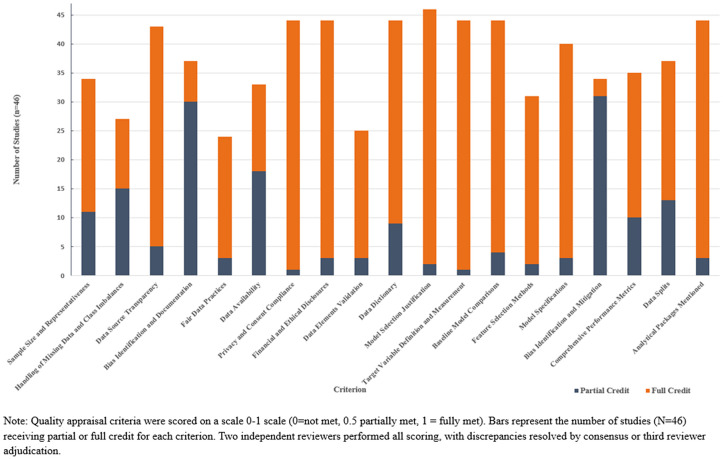
Approach to rating quality and reporting of A-based diagnosis approaches across included studies (N = 46).

### Inter- and intra-rater reliability

Reliability was variably reported. Where provided, agreement between AI outputs and reference readers or between human readers improved or remained high [36,39,42,53–56,74,77,78]. Several studies did not quantify AI-specific reliability despite using expert labels [35,37,38,40,41,43,49,51,52,68].

### Errors and misclassification

Common error patterns included false positives from artifacts or degenerative changes and false negatives for subtle or small lesions [59,74]. Grading confusion at class boundaries was recurrent for lumbar disc categories and early sacroiliitis grades [37,54,58], with misclassification more frequent for intermediate grades or external datasets [53,54,64]. Dataset size and class imbalance affected performance [50,54,64], and moderate AUCs were observed for radiomics models in active sacroiliitis [71]. A prospective LLM evaluation showed substantial diagnostic and management errors across several spinal conditions [63].

### Validation approach

The majority of included studies used retrospective designs in which algorithms were developed and tested on previously collected imaging datasets. A smaller subset employed prospective designs [39–43,57,63,70,75,79] or included external validation cohorts [45,46,53–55,58,59,64]. External validation was performed in 53% of included studies, often with performance attenuation relative to internal splits; across sacroiliitis/BME, LDH/LCCS/LNRC, fracture, and multi-feature grading [45,46,53–55,58,59,64]. Cross-validation frameworks included five-fold and ten-fold schemes [36,57,69], and one study reported internal plus prospective validation [46]. Many reports remained single center with internal splits only [35,37,38,41,42,48,49,72].

### Impact on clinicians and workflow integration

Several papers suggested potential to match or exceed clinician performance or to reduce workload and variability, though most did not quantify downstream clinical impact [36,38,45,46,54,55,62,64]. Time savings or efficiency gains were reported with AI-assisted interpretation or accelerated acquisition [52,78,79]. Nevertheless, almost all systems remained research-only without routine clinical deployment [35,37,39,41–44,49–51,53,56–60,64–66,68,70,72,74,77].

### Usability, interpretability, and implementation considerations

Most of the included studies are proof-of-concept or research-only, with no studies reporting routine clinical deployment. Nearly all systems were evaluated within research pipelines, and authors consistently noted further validation would be required prior to clinical integration.

Interpretability methods included feature importance (Gini) and probability outputs [35,60] saliency/heat-map techniques (Grad-CAM or integrated gradients) [45,55,57,74], and transparent geometric metrics for stenosis [36]. Several models remained “black-box” CNNs with limited explainability [46,50,64]. Some facilitators that were noted included improved image quality and reliability [77,78], potential picture archiving sand communication systems (PACS) integration or clinical net benefit [36,38], open-source release [57], and regulatory clearance for the underlying reconstruction tool [79]. Barriers included single-center training, lack of external validation, protocol heterogeneity, class imbalance, limited demographics, and the need for standardized acquisition and labeling [35,38,57,59,64,67,74].

### Quality assessment ratings

Across the 46 studies evaluated, reporting quality was generally strong for criteria related to privacy and consent compliance, financial and ethical disclosures, target variable definition, model justification, and baseline model comparisons, which were consistently well documented. Most studies also provided clear analytical package descriptions and demonstrated reasonable transparency and data documentation. However, notable deficiencies were observed in the handling of missing data and class imbalances, data element validation, model specification, and feature selection methods, each of which were addressed fully in fewer than half of the studies. Moderate reporting quality was evident for domains such as data availability, bias identification and mitigation, and data splitting procedures. These findings are summarized in Fig 2, which depicts the number of studies receiving full or partial credit for each reporting criterion.

## Discussion

This scoping review provides an overview of recent applications of artificial intelligence for the diagnosis of spinal disorders. Across 46 included studies, the majority reported on imaging-based models, most often applied to MRI, CT, or radiographs. A smaller number incorporated clinical data or combined clinical and imaging inputs. The emphasis on imaging aligns with broader trends in medical AI research, where computer vision techniques dominate because of the relative availability of structured image datasets [31,80].

Many studies reported high diagnostic performance, with authors describing accuracies that approached or matched clinician benchmarks, across various applications including, but not limited to, lumbar disc herniation, spinal stenosis, vertebral fractures, scoliosis, and inflammatory disorders (i.e., axial spondyloarthritis). However, despite encouraging performance metrics, important limitations temper these findings. Most studies were retrospective, single-center analyses with small-to-moderate sample sizes. Few studies included external validation, and prospective testing in clinical workflows was rare. The lack of external testing reduces confidence in generalizability and is consistent with limitations reported in other AI reviews in spine care [30,81].

The diagnostic targets were heterogeneous, reflecting both the versatility of AI methods and the fragmented state of the literature. Some studies focused on binary classification of pathology, while others attempted severity grading or prognostic prediction. This variation limited direct comparison across studies. A small subset examined the integration of multimodal data, combining imaging with clinical or demographic variables, suggesting that broader data inputs may enhance diagnostic utility. At the same time, several reports lacked transparency in data sources, preprocessing, and feature selection, making reproducibility difficult to assess. These gaps align with concerns raised in prior reviews, which emphasized the need for standardized outcome measures and consistent reporting frameworks [82,83].

An emerging pattern in the literature was that AI was most frequently studied in roles augmenting rather than replacing human interpretation. Several studies reported reduced interobserver variability or improved efficiency when AI tools were applied as decision-support systems alongside clinician review. However, the path from research prototype to clinical deployment involves challenges that were largely unaddressed in the included literature, including regulatory approval requirements, integration with existing clinical infrastructure such as PACS systems, interpretability standards for clinician-facing tools, and the need for prospective evidence in diverse patient populations [84]. Addressing these implementation challenges will be necessary before the diagnostic potential suggested by this literature can be realized in routine care.

Overall, diagnostic AI in spine care shows potential to support earlier detection, improve efficiency, and standardize interpretation across diverse conditions. However, evidence remains preliminary. Several specific evidence gaps identified in this review warrant further investigation. First, the predominance of imaging-based retrospective studies from Asia and Europe means that prospective, multi-center studies in diverse geographic and clinical settings are needed to establish generalizability. Second, the near-absence of non-imaging and multimodal diagnostic tools in the literature represents an underdeveloped area where primary research is warranted, particularly given the potential of combined clinical-imaging approaches demonstrated in axial spondyloarthritis studies. Third, no included studies evaluated real-world clinical deployment or measured patient outcomes associated with AI-assisted diagnosis, representing a critical gap that future prospective studies should address. Fourth, the heterogeneity in outcome definitions, validation approaches, and reporting standards across the current literature suggest that a methodological consensus or reporting framework for AI diagnostic studies in spine care would be a valuable contribution.

Our results provide a snapshot of a rapidly evolving domain. The advancement of AI in healthcare, paralleling its expansion in other sectors, has precipitated substantial concerns regarding data privacy, ethical considerations, and regulatory obstacles. Emerging contentious issues encompass data ownership, model stewardship, data and model bias, as well as model transparency and interpretability [85–89]. These concerns are particularly pertinent to patient privacy, data drift, the potential for model manipulation, biases in models that may unjustly affect marginalized populations, and disparities in access to high-quality care. To address these challenges, guidelines aimed at mitigating risks associated with AI systems have been promulgated by the US Federal Trade Commission [90], the European Union [91], China [92], and various industry and professional stakeholders [93]. As AI becomes increasingly integrated into healthcare delivery and operational processes, discussions regarding the ethical and effective utilization of AI are expected to intensify.

### Limitations

The search was restricted to studies published in English between January 1^st^, 2019, and December 31^st^, 2024, which may have led to the exclusion of relevant work published in other languages or outside this time frame. No formal risk of bias assessment was performed, consistent with the scoping review methodology, although a structured quality appraisal was applied to provide context. Several common sources of bias were identified narratively across the included literature. The predominance of retrospective, single-center designs introduces selection bias and limits the representativeness of training datasets seen in included studies. Small sample sizes were frequently noted, particularly in studies evaluating non-imaging or multimodal models, which increases the risk of overfitting and reduces confidence in reported performance metrics. The reliance on internal validation splits in a substantial number of the studies included in this review.

Underrepresentation of certain populations, settings, and geographic regions is also a notable limitation of the current evidence base. A large portion of included studies were conducted in Asia and Europe, with limited representation from North America, South America, the Middle East, and Africa. No studies were identified from low- or middle-income country settings, which raises questions about the generalizability of findings to healthcare systems with different imaging infrastructure, patient demographics, and resource availability. Within studies, demographic reporting was frequently incomplete, with many studies not reporting patient age distributions, sex, race, or comorbidity profiles. This limits the ability to assess whether AI models perform equitably across patient subgroups, which is a recognized concern in medical AI research.

There was substantial heterogeneity across included studies in terms of AI model design, data inputs, diagnostic targets, and often without standardized thresholds or reporting conventions. This degree of methodological variation meant that performance metrics were not directly comparable across studies, and pooled quantitative analysis was not appropriate.

Most included studies were retrospective and often single center, which may affect generalizability. Details regarding data sources, preprocessing, and model development were not consistently reported, making reproducibility difficult to assess. In addition, many of the identified AI applications were evaluated in narrowly defined patient groups. The absence of multi-center or prospective validation methods within the included studies indicates that clinical applicability is uncertain.

## Conclusions

We present a comprehensive overview of the literature on the application of AI in the diagnosis of spinal conditions. Studies frequently reported AI system performance comparable to or supportive of human interpretation. However, methodological variability, reporting transparency limitations, and reliance on retrospective single-center datasets were common. Based on the evidence mapped in this review, future studies should prioritize prospective and multi-center designs, adopt standardized reporting frameworks such as TRIPOD-AI [33], utilize diverse patient populations with complete demographic reporting, and evaluate AI tools within clinical workflows rather than isolated research pipelines. Lastly, investment in the infrastructure needed to support prospective AI validation, including standardized imaging protocols, data sharing frameworks, and regulatory pathways for AI-based diagnostic tools, will be critical to translating research findings into safe and equitable clinical practice.

## Supporting information

S1 File Supplemental search strategy. (DOCX)

S2 File PRISMA-ScR. (DOCX)
