# Supplementary material for: Artificial intelligence in spine care: A scoping review of diagnostic applications
Source: PLoS One. 2026 Jul 28;21(7):e0352200. doi: 10.1371/journal.pone.0352200 (PMC13411900; doi:10.1371/journal.pone.0352200)
Supplement: S1 File — (DOCX) [file pone.0352200.s001.docx]

**Supplemental 1**

| Ovid MEDLINE(R) ALL | ALL <1946 to January 29, 2025>  https://ovidsp.ovid.com/ovidweb.cgi?T=JS&NEWS=N&PAGE=main&SHAREDSEARCHID=1QIWXoMbNV210d3XzOxy472HMQQH9uvODDWVXGB1N8LOLLZFKNkB6iWEMC0aD4gta  1 [Concept 1: back pain/spine] 0  2 back pain/ or low back pain/ 47094  3 (back pain or low-back pain or back ache or low-back-ache).mp. 80901  4 Sciatica/ 5260  5 sciatic*.mp. 42328  6 dorsalgia.mp. 139  7 ischias.mp. 161  8 ischialgia.mp. 137  9 lumboischialgia.mp. 51  10 radiculalgia.mp. 97  11 spin* pain.mp. 2717  12 exp Spinal Nerve Roots/ 34043  13 (Spinal Nerve Root* or Cauda Equina or Spinal Ganglia).mp. 18948  14 (Radicular or radiculopath$).mp. 20709  15 exp Sacroiliac Joint/ 4883  16 sacroiliac-joint*.mp. 7676  17 spin* dysfunction.mp. 237  18 2 or 3 or 4 or 5 or 6 or 7 or 8 or 9 or 10 or 11 or 12 or 13 or 14 or 15 or 16 or 17 173920  19 [Concept 2: AI] 0  20 artificial intelligence/ or machine learning/ or deep learning/ or neural networks, computer/ 160580  21 artificial intelligence.mp. 90123  22 AI.ti,ab. 65508  23 machine learning.mp. 145616  24 deep learning.mp. 80548  25 Natural Language Processing/ 7590  26 natural language processing.mp. 14560  27 augmented reality.mp. 6199  28 neural net*.mp. 138407  29 computation.mp. 45705  30 Clinical Reasoning/ or reasoning.mp. 32975  31 20 or 21 or 22 or 23 or 24 or 25 or 26 or 27 or 28 or 29 or 30 460151  32 [Combine Concepts 1 and 2] 0  33 18 and 31 966  34 [Project 1 (Concept 3A): Diagnosis] 0  35 exp Diagnosis/ or diagnos*.mp. 12331589  36 electrodiagnosis.mp. 7542  37 clinical assessment.mp. 33868  38 medical history.mp. 70838  39 exp Tomography, X-Ray Computed/ or computed tomography.mp. 703484  40 x-ray*.ti,ab. 459799  41 CT scan*.ti,ab. 127848  42 magnetic resonance imag*.mp. or exp Magnetic Resonance Imaging/ 701568  43 MRI.ti,ab. 342861  44 exp Diagnostic Imaging/ 3025242  45 imaging.mp. 2634443  46 screening.mp. 842475  47 Point-of-Care Systems/ or exp decision support systems, clinical/ or "predictive analytics".mp. 29162  48 (bedside comput* or bedside technolog* or point-of-care).mp. 47538  49 "Predictive Value of Tests"/ 231153  50 predictive value.mp. 326077  51 exp Physical Examination/ 1423211  52 physical exam*.mp. 121435  53 Laboratory.mp. 700996  54 exp Risk Assessment/ 327322  55 Risk Assessment*.mp. 389048  56 exp Nuclear Medicine/ or nuclear medicine.mp. 20565  57 Positron Emission Tomography.mp. or exp Positron-Emission Tomography/ 127296  58 PET scan*.mp. 13476  59 Fluoroscopy.mp. or exp Fluoroscopy/ 39898  60 exp Radiography/ or Radiography.mp. 1262306  61 Magnetic Resonance Spectroscopy.mp. or exp Magnetic Resonance Spectroscopy/ 238063  62 Electromyography.mp. or exp Electromyography/ 100406  63 Nerve Conduction Studies/ 52  64 Nerve Conduction Stud*.mp. 7122  65 exp Neuroimaging/ 205163  66 Neuroimag*.mp. 89919  67 exp Biopsy/ 315768  68 Biops*.mp. 624289  69 Blood Chemical Analysis.mp. or exp Blood Chemical Analysis/ 145862  70 exp Genetic Testing/ 57025  71 Genetic Test*.mp. 76270  72 exp Molecular Diagnostic Techniques/ 22092  73 Molecular Diagnostic Technique*.mp. 15338  74 Histopathology.mp. 91597  75 Bone Density Test*.mp. 287  76 exp Medical History Taking/ 22776  77 ((patient or medical) adj2 history taking).mp. 21131  78 exp Clinical Decision-Making/ 18709  79 (clinical adj2 (decision* or reason*)).mp. 81540  80 exp Health Status Indicators/ 355947  81 Health Status.mp. 191381  82 Wearable Electronic Devices/ 10559  83 (wearable adj2 (sensor* or device* or tracker*)).mp. 23484  84 Digital Pathology.mp. 2990  85 Precision Medicine Diagnos*.mp. 31  86 or/35-85 14430047  87 18 and 31 and 86 721  88 limit 87 to yr="2019 -Current" 513 |
| --- | --- |
| AMED (Allied and Complementary Medicine) | <1985 to December 2024>  https://ovidsp.ovid.com/ovidweb.cgi?T=JS&NEWS=N&PAGE=main&SHAREDSEARCHID=2UDYTksJ9HnBdsR8XDq58fCJ9hM9Ukfmb6wynpmyU0jxdDVwFlLnH9knm6vkREfoG  1 [Concept 1: back pain/spine] 0  2 back pain/ or low back pain/ 5309  3 (back pain or low-back pain or back ache or low-back-ache).mp. 7665  4 Sciatica/ 182  5 sciatic*.mp. 577  6 dorsalgia.mp. 3  7 ischias.mp. 3  8 ischialgia.mp. 4  9 lumboischialgia.mp. 1  10 radiculalgia.mp. 0  11 spin* pain.mp. 231  12 exp Spinal Nerve Roots/ 102  13 (Spinal Nerve Root* or Cauda Equina or Spinal Ganglia).mp. 204  14 (Radicular or radiculopath$).mp. 477  15 exp Sacroiliac Joint/ 237  16 sacroiliac-joint*.mp. 392  17 spin* dysfunction.mp. 52  18 2 or 3 or 4 or 5 or 6 or 7 or 8 or 9 or 10 or 11 or 12 or 13 or 14 or 15 or 16 or 17 9023  19 [Concept 2: AI] 0  20 (machine learning or deep learning or neural network$1).mp. 400  21 artificial intelligence.mp. 74  22 AI.ti,ab. 169  23 machine learning.mp. 141  24 deep learning.mp. 37  25 Natural Language Processing.mp. 9  26 natural language processing.mp. 9  27 augmented reality.mp. 32  28 neural net*.mp. 251  29 computation.mp. 110  30 Clinical Reasoning/ or reasoning.mp. 894  31 20 or 21 or 22 or 23 or 24 or 25 or 26 or 27 or 28 or 29 or 30 1645  32 [Project 1 (Concept 3A): Diagnosis] 0  33 exp Diagnosis/ or diagnos*.mp. 82320  34 electrodiagnosis.mp. 306  35 clinical assessment.mp. 5608  36 medical history.mp. 731  37 exp Tomography, X-Ray Computed/ or computed tomography.mp. 1365  38 x-ray*.ti,ab. 2262  39 CT scan*.ti,ab. 480  40 magnetic resonance imag*.mp. or exp Magnetic Resonance Imaging/ 3019  41 MRI.ti,ab. 1604  42 exp Diagnostic Imaging/ 8319  43 imaging.mp. 6571  44 screening.mp. 4834  45 (Point-of-Care System$1 or decision support or "predictive analytics").mp. 81  46 (bedside comput* or bedside technolog* or point-of-care).mp. 48  47 "Predictive Value of Tests"/ 970  48 predictive value.mp. 1476  49 exp Physical Examination/ 888  50 physical exam*.mp. 1626  51 Laboratory.mp. 5002  52 Risk Assessment.mp. 398  53 Risk Assessment*.mp. 428  54 exp Nuclear Medicine/ or nuclear medicine.mp. 30  55 Positron Emission Tomography.mp. or exp Positron-Emission Tomography/ 105  56 PET scan*.mp. 16  57 Fluoroscopy.mp. or exp Fluoroscopy/ 292  58 exp Radiography/ or Radiography.mp. 3567  59 Magnetic Resonance Spectroscopy.mp. or exp Magnetic Resonance Spectroscopy/ 132  60 Electromyography.mp. or exp Electromyography/ 5813  61 Nerve Conduction.mp. 359  62 Nerve Conduction Stud*.mp. 174  63 Neuroimaging.mp. 310  64 Neuroimag*.mp. 315  65 Biops*.mp. 1149  66 Blood Chemical Analysis.mp. or exp Blood Chemical Analysis/ 3  67 Genetic Test$.mp. 50  68 Genetic Test*.mp. 50  69 Molecular Diagnostic Technique*.mp. 1  70 Histopathology.mp. 393  71 Bone Density Test*.mp. 7  72 exp Medical History Taking/ 386  73 ((patient or medical) adj2 history taking).mp. 405  74 Clinical Decision-Making.mp. 595  75 (clinical adj2 (decision* or reason*)).mp. 1492  76 Health Status Indicator$.mp. 11  77 Health Status.mp. 4575  78 (Wearable adj2 Device$).mp. 172  79 (wearable adj2 (sensor* or device* or tracker*)).mp. 244  80 Digital Pathology.mp. 2  81 Precision Medicine Diagnos*.mp. 0  82 or/33-81 98367  83 18 and 31 and 82 50  84 limit 83 to yr="2019 -Current" 12 |
| Cochrane CENTRAL ID  n=31 | (artificial intelligence OR machine learning OR deep learning OR neural network) AND (back pain OR low back pain OR lumbar pain) AND (diagnosis OR detection OR identification) with Publication Year from 2019 to 2025, in Trials 31 |
| Embase | <1974 to 2025 January 29>  https://ovidsp.ovid.com/ovidweb.cgi?T=JS&NEWS=N&PAGE=main&SHAREDSEARCHID=2UDYTksJ9HnBdsR8XDq58edDyC6L0Z5kekIf6AQZmEaCer7ucGoCqN8BDKuVvznfz  1 [Concept 1: back pain/spine] 0  2 exp backache/ 147254  3 (back pain or low-back pain or back ache or low-back-ache).mp. 122495  4 Sciatica/ 3419  5 sciatic*.mp. 46947  6 dorsalgia.mp. 239  7 ischias.mp. 64  8 ischialgia.mp. 4802  9 lumboischialgia.mp. 75  10 radiculalgia.mp. 146  11 spin* pain.mp. 6613  12 exp Spinal Root/ 5027  13 (Spinal Nerve Root* or Cauda Equina or Spinal Ganglia).mp. 11971  14 (Radicular or radiculopath$).mp. 30212  15 exp Sacroiliac Joint/ 9102  16 sacroiliac-joint*.mp. 11762  17 spin* dysfunction.mp. 325  18 2 or 3 or 4 or 5 or 6 or 7 or 8 or 9 or 10 or 11 or 12 or 13 or 14 or 15 or 16 or 17 249944  19 [Concept 2: AI] 0  20 artificial intelligence/ or machine learning/ or deep learning/ or artificial neural network/ 304247  21 artificial intelligence.mp. 108088  22 AI.ti,ab. 86364  23 machine learning.mp. 192823  24 deep learning.mp. 94842  25 Natural Language Processing/ 14748  26 natural language processing.mp. 17479  27 augmented reality.mp. 7209  28 neural net*.mp. 173243  29 computation.mp. 47010  30 Clinical Reasoning/ or reasoning.mp. 42379  31 20 or 21 or 22 or 23 or 24 or 25 or 26 or 27 or 28 or 29 or 30 559166  32 [Combine Concepts 1 and 2] 0  33 18 and 31 1681  34 [Project 1 (Concept 3A): Diagnosis] 0  35 exp Diagnosis/ or diagnos*.mp. 11626452  36 electrodiagnosis.mp. 7867  37 clinical assessment.mp. 304971  38 medical history.mp. 284764  39 exp x-ray computed tomography/ or computed tomography.mp. 735131  40 x-ray*.ti,ab. 504315  41 CT scan*.ti,ab. 227254  42 magnetic resonance imag*.mp. or exp nuclear magnetic resonance imaging/ 1392709  43 MRI.ti,ab. 585436  44 exp Diagnostic Imaging/ 282210  45 imaging.mp. 2652791  46 screening.mp. 1452235  47 Point-of-Care System/ or exp decision support system/ or "predictive analytics".mp. 45224  48 (bedside comput* or bedside technolog* or point-of-care).mp. 64002  49 Predictive Value/ 279677  50 predictive value.mp. 353527  51 exp Physical Examination/ 350794  52 physical exam*.mp. 363379  53 Laboratory.mp. 1191660  54 exp Risk Assessment/ 781051  55 Risk Assessment*.mp. 827611  56 exp Nuclear Medicine/ or nuclear medicine.mp. 56390  57 Positron Emission Tomography.mp. or exp Positron-Emission Tomography/ 274076  58 PET scan*.mp. 37595  59 Fluoroscopy.mp. or exp Fluoroscopy/ 80827  60 exp Radiography/ or Radiography.mp. 1413839  61 Magnetic Resonance Spectroscopy.mp. or exp nuclear magnetic resonance spectroscopy/ 158803  62 Electromyography.mp. or exp Electromyography/ 109102  63 Nerve Conduction Studies/ or electroneurography/ 3484  64 Nerve Conduction Stud*.mp. 12863  65 exp Neuroimaging/ 206116  66 Neuroimag*.mp. 243176  67 exp Biopsy/ 978508  68 Biops*.mp. 1209819  69 Blood Chemical Analysis.mp. or exp Blood chemistry/ 89418  70 exp Genetic screening/ 139403  71 Genetic Test*.mp. 74201  72 exp Molecular Diagnosis/ 32878  73 Molecular Diagnostic Technique*.mp. 1153  74 Histopathology.mp. 898852  75 Bone Density Test*.mp. 453  76 exp Medical History Taking/ 278892  77 ((patient or medical) adj2 history taking).mp. 1137  78 exp Clinical Decision-Making/ 78702  79 (clinical adj2 (decision* or reason*)).mp. 148461  80 exp Health Status Indicator/ 53729  81 Health Status.mp. 210448  82 Wearable Device/ 4786  83 (wearable adj2 (sensor* or device* or tracker*)).mp. 23332  84 Digital Pathology.mp. 4342  85 Precision Medicine Diagnos*.mp. 43  86 or/35-85 15333238  87 18 and 31 and 86 1328  88 limit 87 to yr="2019 -Current" 956 |
| Web of Science  n=342 citations | https://www.webofscience.com/wos/woscc/summary/9d9b035b-b400-47be-9cb1-87013bec384b-01468ad0f2/relevance/1  TS=("artificial intelligence" OR "machine learning" OR "deep learning" OR "neural network*") AND TS=("back pain" OR "low back pain" OR "lumbar pain") AND TS=(diagnos* OR detection OR identification) |
| SCOPUS  n=608 citations | TITLE-ABS-KEY ( ( "artificial intelligence" OR "machine learning" OR "deep learning" OR "neural network*" ) ) AND TITLE-ABS-KEY ( ( "diagnosis" OR "diagnostic" OR "detection" OR "screening" OR "assessment" ) ) AND TITLE-ABS-KEY ( ( "back pain" OR "backache" OR "spinal pain" OR "low back pain" OR "lumbar pain" OR "disc herniation" OR "sciatica" ) ) AND PUBYEAR > 2018 AND PUBYEAR < 2026 |
